# VDAC2 enables BAX to mediate apoptosis and limit tumor development

**DOI:** 10.1038/s41467-018-07309-4

**Published:** 2018-11-26

**Authors:** Hui San Chin, Mark X. Li, Iris K. L. Tan, Robert L. Ninnis, Boris Reljic, Kristen Scicluna, Laura F. Dagley, Jarrod J. Sandow, Gemma L. Kelly, Andre L. Samson, Stephane Chappaz, Seong L. Khaw, Catherine Chang, Andrew Morokoff, Kerstin Brinkmann, Andrew Webb, Colin Hockings, Cathrine M. Hall, Andrew J. Kueh, Michael T. Ryan, Ruth M. Kluck, Philippe Bouillet, Marco J. Herold, Daniel H. D. Gray, David C. S. Huang, Mark F. van Delft, Grant Dewson

**Affiliations:** 1 Walter and Eliza Hall Institute of Medical Research, 1G Royal Parade, Parkville Melbourne, VIC 3052 Australia; 2 Department of Medical Biology, University of Melbourne, Parkville Melbourne, VIC 3010 Australia; 3 Department of Anatomy and Developmental Biology, Monash Biomedicine Discovery Institute, Monash University, Victoria, 3800 Australia; 4 Department of Surgery, The University of Melbourne, Parkville, 3010 Australia; 5 Department of Neurosurgery, Royal Melbourne Hospital, Parkville, 3050 Australia; 6 Department of Biochemistry and Molecular Biology, Monash University, Melbourne, VIC 3800 Australia

## Abstract

Intrinsic apoptosis is critical to prevent tumor formation and is engaged by many anti-cancer agents to eliminate tumor cells. BAX and BAK, the two essential mediators of apoptosis, are thought to be regulated through similar mechanisms and act redundantly to drive apoptotic cell death. From an unbiased genome-wide CRISPR/Cas9 screen, we identified VDAC2 (voltage-dependent anion channel 2) as important for BAX, but not BAK, to function. Genetic deletion of *VDAC2* abrogated the association of BAX and BAK with mitochondrial complexes containing VDAC1, VDAC2, and VDAC3, but only inhibited BAX apoptotic function. Deleting *VDAC2* phenocopied the loss of *BAX* in impairing both the killing of tumor cells by anti-cancer agents and the ability to suppress tumor formation. Together, our studies show that efficient BAX-mediated apoptosis depends on VDAC2, and reveal a striking difference in how BAX and BAK are functionally impacted by their interactions with VDAC2.

## Abstract (web summary)

BAX and BAK are pro-apoptotic proteins whose activity is essential for the action of many anti-cancer drugs and to suppress tumorigenesis. Here, the authors perform a genome-wide CRISPR/Cas9 screen and identify VDAC2 as a promoter of BAX-mediated apoptosis that is important for an efficient chemotherapeutic response and to suppress tumor formation.

## Introduction

Apoptotic cell death is a fundamental process that is essential for embryonic development and immune system homeostasis. BAX and BAK are members of the BCL-2 family of proteins that have essential, but redundant, functions as mediators of intrinsic apoptosis^1–3^. The activation of BAX and BAK and their consequent self-association permeabilizes the mitochondrial outer membrane (MOM) to instigate cytochrome *c* release and cell death^1^. While BAK is predominantly integrated into the MOM, BAX is predominantly cytosolic. Their distinct subcellular localizations may reflect different rates of retrotranslocation from the MOM to the cytosol^4–6^, although the precise determinants of their recruitment to the MOM to mediate cell killing are unclear. Many chemotherapeutic agents indirectly trigger BAX/BAK-mediated apoptosis whereas BH3-mimetic compounds, such as venetoclax (ABT-199), directly inhibit BCL-2 proteins to drive apoptosis^2,7,8^. Venetoclax, which selectively targets BCL-2, has proven highly efficacious for patients with high-risk chronic lymphocytic leukemia (CLL) leading to its approval for treating such patients^9^.

The VDAC channels (VDAC1, VDAC2, and VDAC3) are responsible for the transport of low molecular weight metabolites across the MOM including adenosine triphosphate (ATP) and adenosine diphosphate (ADP). Early studies suggested that the VDACs were responsible for the release of cytochrome *c* across the MOM^10^. However, that cells devoid of all three VDAC isoforms could still undergo apoptosis argued against such a role^11^. Instead, VDACs have been proposed to influence apoptosis by interacting with BCL-2 family proteins including BCL-X_L_, BAX, and BAK^12–15^. In this regard, the prevailing dogma is that VDAC2 acts to limit apoptosis by sequestering BAK^16^.

In marked contrast to this, we identified VDAC2 in an unbiased genome-wide screen for factors required for BAX to function. In the absence of *VDAC2*, cell killing mediated by BAX, but not BAK, is abolished. Moreover, the interaction with VDAC2 is critical for BAX to mediate cell death in response to chemotherapeutic agents both in vitro and in vivo, as well as for BAX to limit tumor development in a cMyc-driven model of acute myeloid leukemia (AML). Our genetic and functional studies unequivocally define a critical and unique requirement for VDAC2 in the apoptotic activity of BAX.

## Results

### VDAC2 is required for BAX to mediate apoptosis

**Fig. 1:**
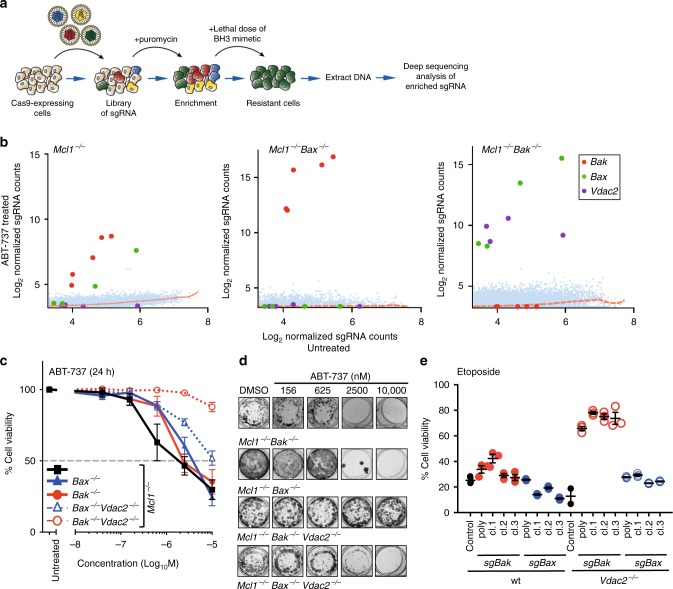
CRISPR/Cas9 screen identifies VDAC2 as a promoter of BAX-mediated apoptosis. **a** Outline of the genome-wide CRISPR/Cas9 library screens to identify mediators of intrinsic apoptosis. **b** VDAC2 promotes BAX apoptotic function. MEFs expressing Cas9 and a whole-genome sgRNA library were treated with ABT-737 (LC90, 250 nM, see Supplementary Fig. 1b) for 48 h. Resistant cells were recovered after 5 days and enriched sgRNAs identified by deep-sequencing. Plots show independent sgRNAs in ABT-737 treated versus untreated controls cells collated and normalized from 2 to 4 independent experiments. Dashed lines represent LOWESS regression curves fitted to the data. **c** Deletion of *Vdac2* protects from BAX-mediated apoptosis in response to ABT-737. Clones (*Mcl1*^*−/−*^*Bax*^*−/−*^*Vdac2*^*−/−*^ and *Mcl1*^*−/−*^*Bak*^*−/−*^*Vdac2*^*−/−*^) or polyclonal populations (*Mcl1*^*−/−*^*, Bax*^*−/−*^ and, *Bak*^*−/−*^) of MEFs were treated with escalating doses of ABT-737 for 24 h and cell viability assessed by PI exclusion. Data are mean+/− SEM of at least three independent experiments with 3–4 independent clones. **d** Deletion of *Vdac2* provides long-term protection from BAX-mediated cell death. MEFs of the indicated genotype were treated with the indicated concentration of ABT-737 and colony formation was assessed after 5 days. **e** Deletion of *Bak* protects *Vdac2*^*−/−*^ MEFs from etoposide-induced apoptosis. Polyclonal populations or three independent MEF clones of the indicated genotype (all on 129sv;C57BL/6 background) were treated with etoposide (10 μM for 24 h) and cell viability assessed by PI exclusion. Data are mean+/− SEM shown for three independent experiments

To identify novel regulators of apoptosis, we undertook unbiased, genome-wide CRISPR/Cas9 library screens (Fig. 1a). *Mcl1*-deficient mouse embryonic fibroblasts (MEFs) were used as they readily undergo BAX/BAK-dependent apoptosis when the remaining pro-survival proteins they express (BCL-2, BCL-X_L_, BCL-W) are inhibited by the BH3-mimetic ABT-737 (Supplementary Fig. 1a-c)^17^. *Mcl1*^−/−^ MEFs stably expressing Cas9 were infected with a genome-wide single guide RNA (sgRNA) library (Supplementary Fig. 1d, 87,897 sgRNAs targeting 19,150 mouse genes^18^). Following treatment with ABT-737, surviving cells were harvested and enriched sgRNAs (relative to untreated MEFs) were identified by deep sequencing (Supplementary Fig. 1e). As expected, sgRNAs targeting *Bax* or *Bak* were enriched in *Mcl1*^−/−^ MEFs that survived ABT-737 treatment (Fig. 1c, Supplementary Table 1).

To identify factors that may act specifically on BAX or BAK, we undertook screens in cells lacking either one of these cell death mediators. *Mcl1*^−/−^*Bax*^−/−^ MEFs were employed to genetically isolate and identify genes required for BAK-driven apoptosis, but we failed to identify any such genes as sgRNAs targeting *Bak* were the only ones over-represented in this screen (Fig. 1b, Supplementary Table 2). Of note, we would not have identified regulators that are critical for normal cell growth or those that act redundantly to facilitate BAK function. Conversely, when we performed the screen using *Mcl1*^−/−^*Bak*^−/−^ MEFs to genetically isolate BAX-dependent apoptosis, eight sgRNAs were significantly enriched; four targeting *Bax* and four targeting *Vdac2* (Fig. 1b, Supplementary Table 3). This indicated that deletion of *Vdac2* protected cells from BAX-mediated apoptosis, but not BAK-mediated apoptosis.

To validate the findings of the screen, we deleted *Vdac2* in either *Mcl1*^−/−^*Bak*^−/−^ or *Mcl1*^−/−^*Bax*^−/−^ MEFs using CRISPR/Cas9 gene editing (Supplementary Fig. 2a). When killing could only be mediated by BAX, deleting *Vdac2* from multiple independently derived *Mcl1*^−/−^*Bak*^−/−^ cells made them highly resistant to ABT-737 (Fig. 1c). By contrast, BAK-mediated killing could proceed in the absence of VDAC2 (Fig. 1c). *Mcl1*^−/−^ MEFs are also killed by the selective inhibition of BCL-X_L_ with A1331852^19^ (Supplementary Fig. 2b). Deleting *Vdac2* likewise protected *Mcl1*^−/−^*Bak*^−/−^ cells, but not *Mcl1*^−/−^*Bax*^−/−^ cells from this BH3-mimetic (Supplementary Fig. 2b), thus demonstrating that protection was not specific to ABT-737 treatment.

Most remarkably, the combined loss of BAK and VDAC2 (but not BAX and VDAC2) also afforded long-term protection against ABT-737 (Fig. 1d) and A1331852 (Supplementary Fig. 2c) in clonogenic assays. Moreover, VDAC2 was important for BAX, but not BAK, to drive apoptosis in response to etoposide treatment (Fig. 1e, Supplementary Figs. 2d, e), indicating that the critical role for VDAC2 in BAX function held true for DNA-damage-induced apoptosis.

It has recently been proposed that BAX and BAK are preferentially activated by BIM and BID respectively^20^. As BIM and BID levels were not significantly altered in VDAC2-deficient MEFs (Supplementary Fig. 2f), changes in these BH3-only proteins were unlikely to account for the resistance to apoptosis observed upon VDAC2 loss. Collectively, our data strongly indicate that apoptosis of cells mediated by BAX, but not BAK, requires VDAC2.

Previous studies have indicated that recombinant BAX can mediate cytochrome *c* release from *Vdac2*^−/−^ mitochondria, although this study did not exclude a role for the residual BAK population in the recruitment of BAX and subsequent MOMP^15^. We analysed cytochrome *c* release mediated by recombinant BAX from mitochondrial fractions isolated from either *Bax/Bak* DKO MEFs or *Bax/Bak/Vdac2* TKO MEFs (Supplementary Fig. 3a). Although recombinant BAX could mediate cytochrome *c* release from both mitochondria following cBID treatment, this was reduced in mitochondria lacking VDAC2 (Supplementary Fig. 3b), supporting that VDAC2 promotes BAX recruitment and function even in the context of high concentrations of recombinant BAX.

### BAX associates with mitochondrial VDAC complex

**Fig. 2:**
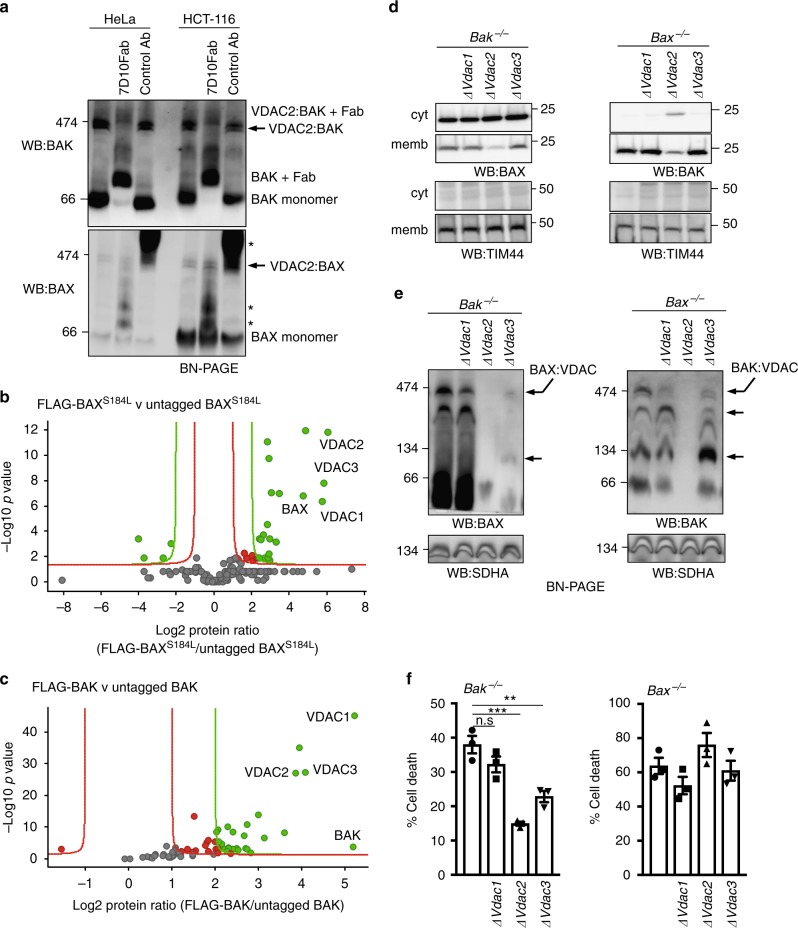
VDAC2 promotes the association of BAX and BAK with a VDAC complex. **a** Endogenous BAX and BAK associate with independent complexes in mitochondria. Mitochondria-enriched fractions from HeLa or HCT116 cells were solubilized in 1% digitonin prior to incubation with a control antibody or an antibody that binds inactive human BAK (7D10), prior to BN-PAGE and immunoblotting for BAK or BAX. * likely cross-reactivity of anti-rat secondary antibody with rat IgG used for gel-shift. Importantly, whilst all of the BAK:VDAC2 complex was gel-shifted by the BAK antibody, the BAX:VDAC2 complex was unaffected. **b** Mass spectrometry analysis of the native BAX complex. Mitochondria from MEFs expressing FLAG-BAX^S184L^ or untagged BAX^S184L^ were solubilized in 1% digitonin prior to anti-FLAG affinity purification and proteins identified by quantitative mass spectrometry analysis. Volcano plot illustrating the log_2_ protein ratios of proteins enriched in the native complex following quantitative pipeline analysis. Proteins were deemed differentially regulated if the log_2_ fold change in protein expression was greater than two-fold (red) or four-fold (green) and a –log_10_ *p* value ≥ 1.3, equivalent to a *p* value ≤ 0.05. **c** Mass spectrometry of the native BAK complex. Mitochondria from MEFs expressing FLAG-BAK or untagged BAK harvested and analyzed as in (**b**). **d** Deletion of VDAC2 impacts mitochondrial localization of BAX and BAK. Clonal populations of *Bax*^*−/−*^ and *Bak*^*−/−*^ MEFs with deleted *Vdac1, Vdac2* or *Vdac3* (denoted ∆) were fractionated into cytosol and membrane and immunoblotted for BAX, BAK or TIM44 as a mitochondrial control. **e** VDAC2 plays the major role in BAX and BAK complex stability. Mitochondria isolated from clonal populations of *Bax*^*−/−*^ and *Bak*^*−/−*^ MEFs with deleted *Vdac1, Vdac2* or *Vdac3* were analyzed on BN-PAGE. Data are representative of two independent clones (see Supplementary Fig. 2e). Intermediate complexes indicated (arrows). **f** BAX-mediated apoptosis is impaired in the absence of VDAC2 and to a lesser extent by VDAC3. Polyclonal populations were treated with etoposide (10 μM) and cell death was assessed by PI uptake. Data are mean+/*−* SEM of three independent experiments. ***p < 0.001; **p < 0.01; n.s, not significant; based on unpaired Student’s *t*-test

Next, we asked whether VDAC2 physically interacts with BAX to promote its activity. We, and others, have reported that BAX and BAK reside in large mitochondrial complexes containing VDAC2 from which they dissociate following the induction of apoptosis^13,14,21^. To determine whether BAX and BAK can associate together in a single complex containing VDAC2, we performed antibody gel-shift assays on mitochondrial fractions prepared from HeLa or HCT116 cells (Fig. 2a). We found that adding Fab fragments of an antibody that binds inactive human BAK (7D10^22^) altered the mobility of all of the BAK:VDAC2 complex on a native gel, whereas the BAX:VDAC2 complex was unaffected (Fig. 2a), indicating that the anti-BAK antibody neither significantly gel-shifts, nor disrupts the BAX-containing complex. Hence, BAK and BAX likely form distinct complexes with VDAC2 on mitochondria.

The large size of these complexes on native gels suggested that they contain other components. To identify constituent proteins, we generated MEFs stably expressing FLAG-BAX^S184L^ or FLAG-BAK (both of which constitutively localize to mitochondria through an interaction with VDAC2^14^) and purified the complexes under native conditions using an anti-FLAG antibody (Supplementary Fig. 4a). Quantitative mass spectrometry analysis of these complexes confirmed the presence of VDAC2 and BAX or BAK, whilst VDAC1 and VDAC3 were also present (Fig. 2b and c, Supplementary Data 1 and 2).

In order to define the role of individual VDACs in forming mitochondrial complexes with BAX or BAK, we next evaluated the impact of deleting each VDAC (Fig. 2d, e, and Supplementary Figs. 4b-d). Deleting *Vdac2* disrupted the mitochondrial localization of both BAX and BAK (Fig. 2d) and abolished their association with mitochondrial complexes (Fig. 2e and Supplementary Fig. 4e). While deleting *Vdac3* did not significantly affect the mitochondrial localization of either BAX or BAK, it did alter the size of the complexes with which BAX and BAK associated (Fig. 2d, e and Supplementary Fig. 4e).

These biochemical studies suggest that the composition of the mitochondrial VDAC:BAX or VDAC:BAK complexes is similar (Fig. 2b, c). VDAC2 was important for BAX and BAK localization on mitochondria and critical for them to associate with these complexes (Fig. 2d, e and Supplementary Fig. 4e). That VDAC3 played an ancillary role (Fig. 2d, e and Supplementary Fig. 4e), suggests a functional hierarchy amongst the VDACs in apoptosis. Consistent with this notion, we found that VDAC2 had the greatest role in BAX-mediated apoptosis, VDAC3 had a lesser role, whilst VDAC1 was largely dispensable (Fig. 2f).

### A distinct surface on VDAC2 drives BAX activity

**Fig. 3:**
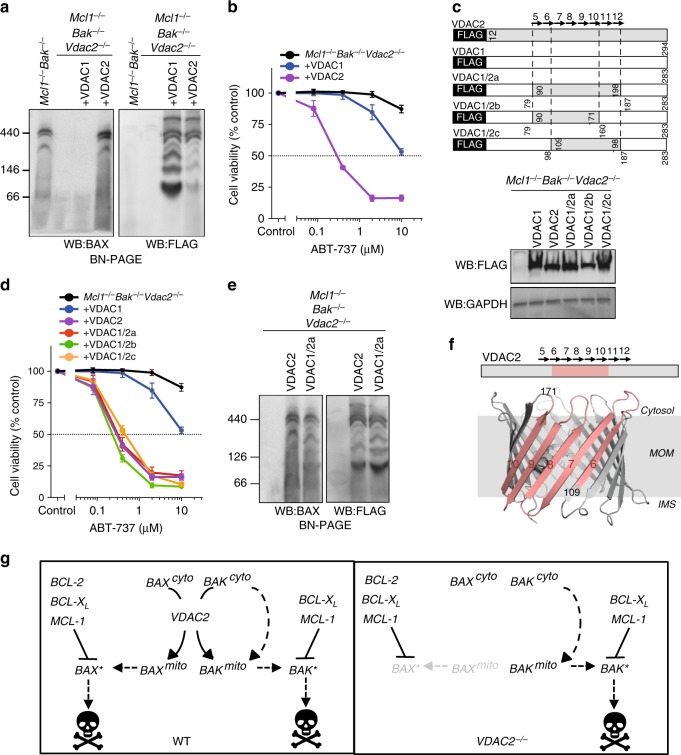
Interaction with VDAC2 is important for BAX apoptotic function. **a** BAX mitochondrial complex formation specifically relies on VDAC2. Mitochondria-enriched fractions from *Mcl1*^*−/−*^*Bak*^*−/−*^*Vdac2*^*−/−*^ MEFs reconstituted with FLAG-mVDAC1 or FLAG-hVDAC2 were analyzed by BN-PAGE and immunoblotted for BAX (left) or FLAG to detect ectopically-expressed VDACs (right). **b** BAX apoptotic function relies on VDAC2. Cells as in (**a**) were treated with ABT-737 and cell viability was assessed. **c**–**e** Rescue of BAX apoptotic function correlates with interaction with a specific region of VDAC2. *Mcl1*^*−/−*^*Bak*^*−/−*^*Vdac2*^*−/−*^ MEFs stably expressing FLAG-mVDAC1/hVDAC2 chimeras (**c**) were analyzed for expression by immunoblotting for FLAG (or GAPDH as a loading control), cell viability following treatment with ABT-737 (**d**), and complex formation by BN-PAGE and immunoblotting for BAX or FLAG (**e**). **f** BAX interacts with a defined region of VDAC2. Interaction of BAX requires aa109-171 of hVDAC2 (salmon) mapped onto the structure of zebrafish VDAC2 (PDB 4BUM^64^). MOM mitochondrial outer membrane, IMS intermembrane space. **g** VDAC2 is essential for BAX, but not BAK to target mitochondria and mediate apoptosis. In wild-type cells, cytosolic BAX (BAX^cyto^) relies on VDAC2 to associate with mitochondria (BAX^mito^) and activate (BAX*). In the absence of VDAC2, BAX cannot drive cell death. Although the ability of BAK to associate with mitochondria is also perturbed in *VDAC2*^*−/−*^ cells^14, 31^, sufficient BAK can still target mitochondria through a VDAC2-independent mechanism to drive apoptosis. Data presented in (**b**) and (**d**) is mean+/*−* SEM of three independent experiments

The clear distinction between VDAC2 and VDAC1 in controlling BAX localization and function allowed us to clarify how VDAC2 might promote the activity of BAX. Re-expressing VDAC2 in VDAC2-deficient cells rescued both BAX complex formation and apoptotic function, whilst expressing VDAC1 did not (Fig. 3a, b). We exploited this difference to map the region on VDAC2 that is necessary to promote BAX activation. Expression of VDAC1/2 chimeras (Fig. 3c and Supplementary Fig. 5), identified a region of VDAC2, comprising central β-strands 7–10, that was sufficient to promote BAX apoptotic function, and support the formation of BAX complexes in the mitochondrial membrane (Fig. 3d–f).

Taken together, we postulate that a distinct surface on VDAC2 recruits BAX to the mitochondria and that disrupting this interaction abrogates BAX function (Fig. 3g). Interestingly, the same region of VDAC2 is reported to interact with BAK^15^, suggesting a conserved mechanism for recruiting both BAX and BAK to mitochondria. However, the impact of *Vdac2* deletion on the apoptotic function of BAX and BAK is strikingly different. That BAK still drives apoptosis without VDAC2 (Figs. 1c, d and 2f), and in some cases even drives enhanced apoptosis^14,16^, indicates that VDAC2 is not the sole conduit for BAK to reach the MOM where it acts (Fig. 3g). Conversely, VDAC2 is essential for BAX recruitment to mitochondria and its absence nullifies BAX apoptotic function (Fig. 3g).

### BAK does not limit embryonic development of *Vdac2*^−/−^ mice

**Fig. 4:**
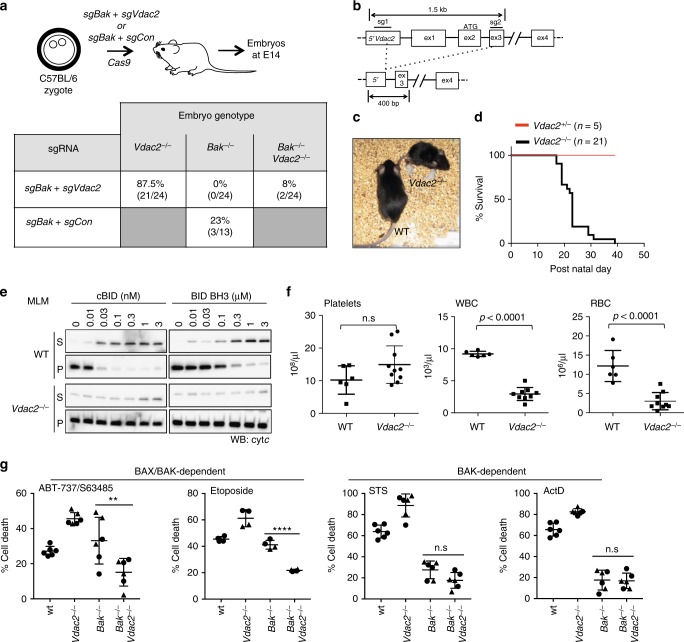
*Vdac2*^*−/−*^ mice do not show evidence of excessive BAK activity. **a** *Bak* deletion is not essential for early embryonic development of *Vdac2*^*−/−*^ mice. C57BL/6J zygotes were injected with DNA encoding Cas9 and sgRNAs targeting *Bak* and *Vdac2* (or *Rosa* as a control) and transplanted into pseudo-pregnant mothers. The percentage embryos at E14.5 with homozygous null alleles are indicated (number of mice in parentheses). **(b)** Gene targeting strategy to generate *Vdac2*^*−/−*^ mice. Targeting by both sgRNAs will result in a deletion of 500 bp whereas targeting by the 3’ sgRNA alone results in indels (see Supplementary Fig. 3a). **c**, **d** *Vdac2*^*−/−*^ mice are runted die post-natally. Kaplan–Meier survival curve of *Vdac2*^*+/−*^ and *Vdac2*^*−/−*^ F0 mice. **e** *Vdac2*^*−/−*^ mitochondria are resistant to MOM permeabilization. Liver mitochondria isolated from age-matched WT and *Vdac2*^*−/−*^ mice were treated with cBID prior to fractionation into supernatant (S) and membrane (P) and immunoblotting for cytochrome *c*. Data are representative of *N* = 2 mice (see Supplementary Fig. 3d). **f** *Vdac2*^*−/−*^ mice show defects in the hematopoietic system. Blood counts for individual age-matched wild-type (WT, N = 6) or *Vdac2*^*−/−*^ (*N* = 9) mice shown with mean+/*−*SD. WBC white blood cells, RBC red blood cells. *P* values calculated by two-tailed Student’s *t*-test. n.s, not significant. **g** Early passage primary *Bak*^*−/−*^ (BR1.4, *triangles;* BR2.1, *circles*), *Vdac2*^*−/−*^ (BV1.1, *triangles;* BV2.4, *circles*), *Bak*^*−/−*^*Vdac2*^*−/−*^ (BV2.1, *triangles*, BV2.3, *circles*) or wild-type (wt, *circles*) MEFs (see Supplementary Figure 8) were treated with etoposide (10 μM), ABT-737 (1 μΜ) and S63485 (1 μM), staurosporine (STS, 1 μM) or actinomycin D (ActD, 1 μM) and cell death assessed by PI uptake after 24 h. Data is mean+/*−*SD of three independent experiments. **p < 0.01; ****p < 0.0001; n.s, not significant based on Students unpaired *t*-test

That our data shows an important role for VDAC2 in promoting BAX-mediated apoptosis was unexpected given that VDAC2 is proposed to inhibit BAK^16^. A proposed consequence of this obligate inhibitory role for VDAC2 is the early embryonic lethality of mice lacking *Vdac2* due to inappropriate and excessive apoptosis driven by unrestrained BAK^16^. Hence, we hypothesized that we would need to co-delete *Bak* to allow *Vdac2*-deficient embryos to survive. To test this hypothesis, we injected C57BL/6J zygotes with plasmid encoding Cas9 together with single guide RNAs targeting *Bak* and *Vdac2* (Fig. 4a). The resulting embryos were harvested at E14.5. Most embryos were *Vdac2*-null (87.5%), yet concomitant *Bak* deletion was only observed in 8% compared with 23% when *Bak* was targeted alone (with sgRNA targeting *Rosa* as a control) (Fig. 4a). This suggested that co-deletion of *Bak* did not promote, nor was it essential for, the survival of *Vdac2*^−/−^ embryos, at least up to the E14.5 developmental stage.

Since a significant number of *Vdac2*^−/−^ embryos survived to E14.5, we set out to determine whether its loss might compromise development later during embryogenesis. *Vdac2*^−/−^ mice were generated on a C57BL/6J genetic background and we allowed the mice to develop to term (Fig. 4b). Of the 26 offspring surviving, 20 were *Vdac2* knock-out with homozygous non-sense (*indels*) mutations (Supplementary Fig. 6a). The absence of VDAC2 protein was confirmed by mass spectrometry or immunoblotting liver extracts from *Vdac2*^−/−^ mice (Supplementary Fig. 6b). Thus, the early embryonic lethality associated with deleting *Vdac2* likely depends on genetic background, being much more severe in 129Sv;C57BL/6 mice^16^ compared to the inbred C57BL/6J strain used here.

### *Vdac2*^−/−^ mice do not exhibit excessive BAK-mediated apoptosis

Although *Vdac2*^−/−^ mice were viable at birth, they failed to gain weight and had to be euthanized by 6 weeks of age because of ill health (Fig. 4c, d and Supplementary Fig. 6c). Regardless, this provided us with an opportunity to investigate the interaction between VDAC2 and BAK in vivo in addition to during embryogenesis, focusing initially on testing whether VDAC2 acts to restrain BAK^16^. Firstly, it is well recognized that BAK-mediates cytochrome *c* release, indicative of MOM damage, when mouse liver mitochondria (MLM) are treated with BH3 peptides such as cBID^23^. This was not enhanced by the deletion of *Vdac2* (Fig. 4e and Supplementary Fig. 6d), but was instead compromised, most probably because BAK levels are reduced in the mitochondria of *Vdac2*-null cells (Fig. 3f and Supplementary Fig. 6e)^14,24^.

Secondly, platelet homeostasis is highly dependent on BAK; its deletion causes elevated platelet counts whereas unrestrained BAK leads to low platelet counts^25^. If VDAC2 acts to restrain BAK in platelets (Supplementary Fig. 7a), deleting VDAC2 should decrease platelet counts, but this was not the case (Fig. 4f). Collectively, our data (Figs. 4a, e, f) suggest that BAK is not necessarily hyperactive in vivo in the absence of VDAC2 and that overt BAK activation is unlikely to account for the premature demise of *Vdac2*^−/−^ mice.

Consistent with this, we found that *Vdac2*-deficient hematopoietic stem cells (HSCs) could function normally upon reconstitution (Supplementary Fig. 7b). Additionally, we found that the absence of VDAC2 did not significantly influence the apoptosis of thymocytes or splenocytes in ex vivo cultures (which can be mediated by either BAX or BAK^26^) (Supplementary Fig. 7c). These data suggest that the overall reduction in white and red blood cell counts in *Vdac2*^−/−^ mice (Fig. 4f) is secondary to their overall ill health rather than due to excessive BAK-mediated apoptosis. Further investigations revealed the likely cause of their early death. *Vdac2*^−/−^ mice had pallid livers (Supplementary Fig. 7d) and their hepatocytes were swollen with central nuclei and clear, distended cytoplasm indicative of cellular edema (Supplementary Fig. 7e). Hydropic swelling often reflects a loss of ionic homeostasis caused by defects in plasma membrane ATP-dependent Na^+^/K^+^ exchange, which can be a consequence of defective mitochondrial ATP production^27^. This liver phenotype would be consistent with the loss of VDAC2 as a transporter of ATP across the MOM, and may explain why *Vdac2*^−/−^ mice fail to thrive.

Although *Vdac2*^−/−^ mice showed no signs of hyperactive BAK, interestingly, those VDAC2-deficient male mice that lived long enough (5 weeks) for such an examination had small testes devoid of mature sperm with expanded numbers of spermatogonia and the appearance of giant cells in seminiferous tubules (Supplementary Fig. 7f). This is a phenotype reminiscent of that observed in *Bax*-deficient mice^28^, and is consistent with our hypothesis that VDAC2 promotes the apoptotic function of BAX. To explore the reliance of BAX on VDAC2 in primary cells, we examined the apoptosis of primary MEFs from embryos that were targeted for *Bak* and/or *Vdac2*. As expected, *Bak* deletion was sufficient to render primary MEFs resistant to stimuli that are largely BAK-dependent (actinomycin D, staurosporine) (Supplementary Fig. 8). However, for apoptotic stimuli that could also be mediated by BAX (etoposide, combined BH3-mimetics), co-deletion of *Vdac2* provided resistance (Supplementary Fig. 8), consistent with BAX being incapable of mediating cell death in the absence of VDAC2. Consistent with Cheng et al.^16^, *Vdac2*^−/−^ primary MEFs were more sensitive to BAK-driven apoptosis (Supplementary Fig. 8). Together, our data highlight the stark difference in the impact of VDAC2 on BAX and BAK apoptotic function, since BAK function is either enhanced or unchanged, whilst BAX apoptotic function is strongly impaired.

### VDAC2 enables BAX to mediate tumor cell killing

**Fig. 5:**
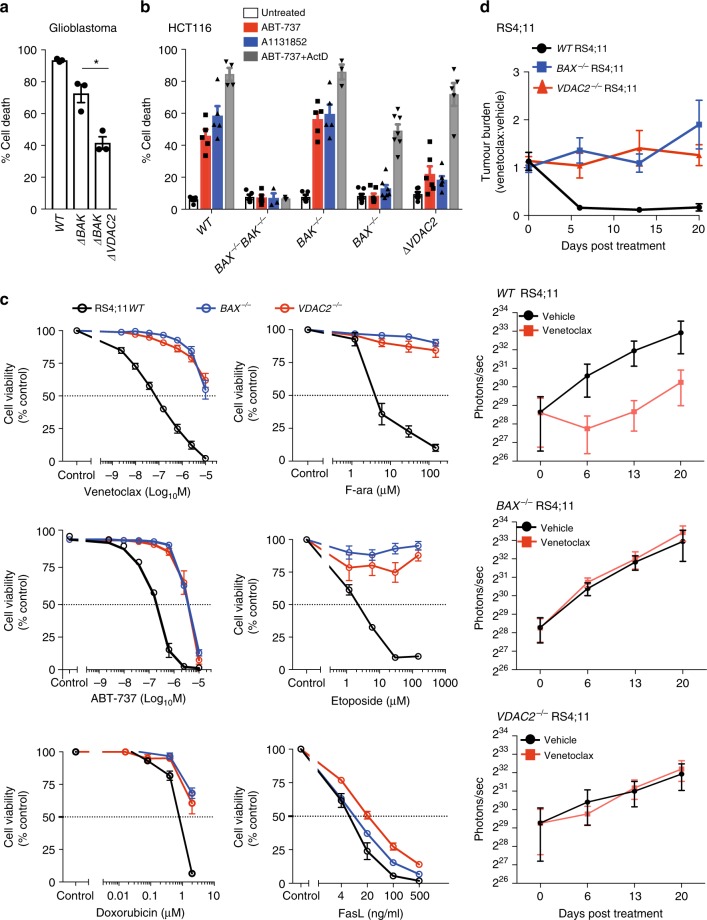
VDAC2 enables BAX-mediated killing of cancer cells in vitro and in vivo. **a** Deletion of *VDAC2* inhibits BAX-mediated apoptosis in glioblastoma cells. Glioblastoma cells (U-251) were treated with ABT-737 (1 μM) and S63845 (1 μM) and cell death assessed after 24 h. Data are mean+/*−*SEM of three independent experiments. *p < 0.05 based on Student’s unpaired *t*-test. **b** Deletion of *VDAC2* protects HCT116 colorectal cancer cells from apoptosis. HCT116 cells were treated with ABT-737 (5 μM), A1331852 (5 μM) or ABT-737 (5 μM) + actinomycin D (1 μM) for 24 h and cell death assessed. Data are mean+/*−*SEM of five independent experiments. **c** BAX or VDAC2 deletion renders RS4;11 acute lymphoblastic leukemia cells resistant to venetoclax or other chemotherapeutic agents. WT, *BAX*^*−/−*^ or *VDAC2*^*−/−*^ RS4;11 cells were treated with venetoclax, ABT-737 and standard-of-care chemotherapies (F-ara, etoposide, doxorubicin) or the BAX/BAK-independent stimulus Fas ligand (FasL) and cell viability assessed by PI exclusion. Data are mean+/*−*SEM of at least 3 independent experiments. **d** Deletion of *VDAC2* renders RS4;11 cells resistant to venetoclax in vivo. RS4;11 cells were subcutaneously engrafted into NOD/SCID/IL-2Rγ^null^ mice, treated with venetoclax and tumor growth monitored by IVIS imaging. The collated data (top panel) is normalized to represent relative tumor burden. Data are mean+/*−* SEM collated from four independent experiments, *N* = 12 mice engrafted with each genotype of RS4;11 cells

Given that our data implicates a central role for VDAC2 in promoting BAX-mediated apoptosis, we hypothesized that the BAX:VDAC2 interaction would also be important for the activity of BAX in tumor cells responding to chemotherapeutic agents. Consistent with this hypothesis, deletion of *VDAC2* in glioblastoma cells engineered to be BAX-dependent (i.e., ∆*BAK*) significantly inhibited apoptosis in response to BH3-mimetics (Fig. 5a and Supplementary Fig. 9a). Due to the inhibitory effect of MCL1 on BAK, the apoptosis of HCT116 colorectal cancer cells in response to either ABT-737^29^ or the BCL-X_L_ inhibitor A1331852 relies on BAX (Fig. 5b). Deletion of *VDAC2* rendered HCT116 cells as resistant to these BH3-mimetic compounds as the loss of BAX alone and remarkably, almost as resistant as the combined loss of BAX and BAK (Fig. 5b and Supplementary Fig. 9b). When apoptosis in the same cells could be also driven by BAK (e.g., combining ABT-737 with actinomycin D treatment), the loss of VDAC2 alone had no impact (Fig. 5b). As in MEFs, there was no significant change in the expression of BIM or BID upon *Vdac2* deletion that might otherwise explain this resistance to apoptosis (Supplementary Fig. 9c).

In the acute lymphoblastic leukemia cell line RS4;11, apoptosis induced by the BCL-2 inhibitor venetoclax (also known as ABT-199)^7,9^ or standard-of-care chemotherapeutic agents is also principally mediated by BAX (Fig. 5c). Independent RS4;11 clones with targeted deletion of *VDAC2* were as resistant to these drugs as *BAX*^−/−^ RS4;11 cells (Fig. 5c and Supplementary Fig. 9d). As expected, BAX- or VDAC2-deficient leukemia cells demonstrated comparable sensitivity to activation of the extrinsic pathway of apoptosis by Fas ligand (Fig. 5c)^30^. To test whether the BAX:VDAC2 axis contributes to the response to BH3-mimetics in vivo, RS4;11 cells expressing a luciferase reporter were engrafted subcutaneously into NOD/SCID/IL-2Rγ^null^ mice. Deletion of either BAX or VDAC2 was sufficient to cause marked resistance to in vivo venetoclax treatment (Fig. 5d). These data establish that VDAC2 is required for BAX-dependent apoptotic responses to cytotoxic drugs in vitro and in vivo.

### VDAC2 enables BAX to limit tumor formation

**Fig. 6:**
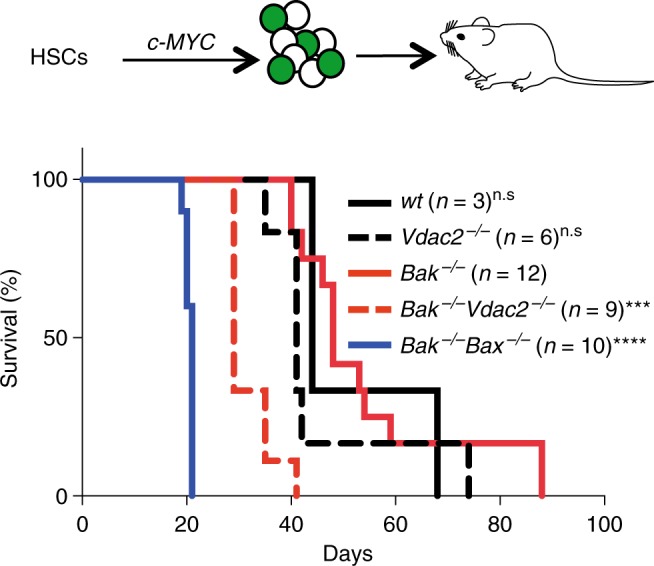
VDAC2 enables BAX to limit tumor development. *Vdac2* deletion accelerates the development of *MYC*-driven AML. Kaplan–Meier survival plot of mice transplanted with wild-type (wt), *Vdac2*^*−/−*^, *Bak*^*−/−*^*, Bak*^*−/−*^*Bax*^*−/−*^ or *Bak*^*−/−*^*Vdac2*^*−/−*^ fetal liver-derived hematopoietic stem cells (HSCs) (2 livers per genotype, see Supplementary Figure 7a) infected with a *c-MYC*–expressing retrovirus. *P* values (Log-rank analysis) of mice injected with *Bak*^*−/−*^ hematopoietic precursors compared with *Bak*^*−/−*^*Vdac2*^*−/−*^ or *Bak*^*−/−*^*Bax*^*−/−*^ precursors are <0.001 (***) and <0.0001 (****) respectively. n.s, not significant

Since the intrinsic apoptotic pathway is an important barrier to tumorigenesis, we hypothesized that the impairment of BAX-mediated apoptosis by deletion of *Vdac2*, when combined with *Bak* deletion, should accelerate tumor development. Fetal liver-derived HSCs derived from *Bak*^−/−^*, Bak*^−/−^*Bax*^−/−^ or *Bak*^−/−^*Vdac2*^−/−^ mice at E14.5 were retrovirally transduced with the *c-MYC* oncogene and transplanted into lethally-irradiated mice to induce AML (Fig. 6 and Supplementary Fig. 8a)^31^. As expected, the combined loss of *Bax* and *Bak* in hematopoietic progenitors accelerated the development of *MYC*-driven AML compared with loss of *Bak* alone (Fig. 6). Strikingly, the combined deletion of *Vdac2* and *Bak* also accelerated AML development when compared with deletion of *Bak* alone (Fig. 6), affirming that VDAC2 is a key mediator of BAX activation in the context of oncogenic stress.

## Discussion

Detailed understanding of how the intrinsic pathway of apoptosis is controlled has paved the way for the development and clinical success of small molecule agonists of the pathway, such as the BCL-2 inhibitor, venetoclax (ABT-199) to treat certain cancers^7,9,32^. BAX and BAK act in a functionally redundant manner to mediate intrinsic apoptosis triggered by venetoclax and also during normal tissue homeostasis. Hence, how these essential cell death mediators are regulated is critical both for understanding normal cell death control and for our efforts to target this pathway for therapeutic benefit.

In this regard, VDAC2 plays an intriguing and controversial role. A previous report suggested that VDAC2 restrains BAK and that the early embryonic lethality observed in *Vdac2*-deficient mice is potentially due to unrestrained BAK activity^16^. However, unlike the mice on a mixed genetic background used in this previous study, we found that *Vdac2*-null mice on an inbred C57BL/6J genetic background were viable at birth, yet they failed to thrive postnatally (Fig. 4c, d). The most prominent pathological finding in these *Vdac2*^−/−^ mice was pallid livers filled with swollen hepatocytes. These edematous cells were likely to have resulted from the loss of normal ionic balance across the plasma membrane by ATP-dependent Na^+^/K^+^ pumps^33,34^. These findings suggest that the metabolite transporter function of VDAC2 is likely important for hepatocyte homeostasis and proper liver function. That *Vdac1*^−/−^ and *Vdac3*^−/−^ mice are viable and do not exhibit such a liver phenotype or premature decline, implies a non-redundant isoform-specific function for VDAC2^16,35,36^.

Definitive conclusions about the role of VDAC2 in controlling apoptosis in vivo are complicated by its dual functions in metabolite transport and in apoptosis and the significant impact of *Vdac2* deletion on the overall health of knockout mice. However, our studies suggest that BAK-mediated apoptosis does not drive the premature lethality of *Vdac2*-null mice: (1) platelet number was not reduced (Fig. 4f); (2) liver mitochondria were not more sensitive to cytochrome *c* release when treated with cBID (Fig. 4e); (3) there was no increase in blood cell apoptosis ex vivo (Supplementary Fig. 7c). That thymocytes isolated from *Vdac2*^−/−^ mice underwent apoptosis ex vivo like those from wild-type mice contrasts with the slightly elevated apoptosis following *Lck-*Cre-mediated deletion of *Vdac2* in thymocytes^37^. Although the reasons for this difference are unclear, Cre-induced toxicity that can confound the analysis of thymocyte apoptosis in this transgenic Cre model^38^ or differences in the developmental stage of *Vdac2* deletion (third CD4/CD8 double negative stage with *Lck*-Cre compared with constitutive in our system) may contribute. In certain settings, such as in primary MEFs, BAK apoptotic activity was elevated in accord with the original report^16^ (see Supplementary Fig. 8b), highlighting that cellular context may contribute to variation in this phenotype. Nevertheless, we consistently observed that while BAK was capable of driving apoptosis without VDAC2, BAX was not.

In accord with other reports^14,21,24^, we confirmed that both BAX and BAK interact with VDAC2 on the MOM through a conserved mechanism (Fig. 3). However, using a range of in vitro and in vivo systems, we discovered that the interaction with VDAC2 is crucial specifically for BAX, but not BAK, to mediate apoptosis. Thus, where BAX is the key mediator of apoptotic cell death, we found identical consequences when either *BAX* or *VDAC2* were genetically deleted. For example, BAX-mediated killing of cancer cells by venetoclax was abrogated in the absence of VDAC2. Consistent with these observations we noted defective spermatogenesis upon deleting *Vdac2*, a phenotype reminiscent of *Bax* loss^28^. An obligate role for the VDACs in apoptosis was previously discounted as MEFs lacking all three isoforms could still undergo apoptotic cell death^11^. Our findings that VDAC2 is important specifically for BAX-mediated apoptosis are reconcilable with this study, given that BAK-mediated apoptosis can proceed in the absence of VDAC2 (Figs. 1c and 2f)^16^, and also in the absence of VDAC1 or VDAC3 (Figs. 1c and 2f). Previous studies using shRNA approaches have implicated a role for VDAC2 in promoting BAX apoptotic activity^14,39^. Our findings using a powerful unbiased screening approach and CRISPR/Cas9 gene editing now indicate that the loss of BAK and VDAC2 can phenocopy the combined loss of BAK and BAX revealing that VDAC2 is important for BAX to initiate apoptosis both in vitro and in vivo.

Given the proposed redundancy between BAX and BAK, and the effect of VDAC2 deletion on the mitochondrial targeting of both BAK and BAX, it is surprising that deleting VDAC2 impairs BAX, but not BAK, apoptotic activity^14,24,39^. This may indicate that VDAC2 is not solely responsible for the localization of BAK to mitochondria. Properties inherent to BAK itself, such as its C-terminal tail or other factors on the MOM could act independently of VDAC2 to promote BAK localization to mitochondria and hence its apoptotic activity (Fig. 3g). Intriguingly, the removal of VDAC2 renders BAK hyper-active in some situations (Supplementary Figure 8b)^14,16^. A possible explanation for the heightened activity of BAK despite its reduced mitochondrial localization in the absence of VDAC2 is that both BAX and BAK need to dissociate from VDAC2 to homo-oligomerize to mediate cytochrome *c* release^13,14^. The residual population of BAK that targets mitochondria independent of VDAC2 does not have to undergo this initial dissociation step, and may be able to directly recruit cytosolic BAK (and cytosolic BAX) to efficiently mediate MOM permeabilization^14^.

Taken together, we have shown that the VDAC2:BAX interaction promotes BAX to mediate apoptosis and this clearly differentiates it from BAK. Thus, we hypothesize that manipulating this interaction may well be therapeutically beneficial in situations where BAX, rather than BAK, is the principal mediator of apoptosis. For example, disrupting or preventing the VDAC2:BAX interaction may be a novel strategy to block BAX activity and hence protect cells in some scenarios from damaging cell death. Disrupting the VDAC2:BAX interaction could be exploited to limit pathological apoptosis following traumatic or ischemic brain injuries since differentiated neurons lack functional BAK^40^. In the context of cancer chemotherapy, BAX is likely the prime driver for certain cytotoxic agents to act (Fig. 5)^41,42^. Our findings are also highly relevant for treatment with venetoclax since its target, BCL-2, principally limits BAX rather than BAK^43^. Venetoclax is now approved in the US and an increasing number of other countries as monotherapy for patients with relapsed/refractory CLL, but primary and secondary resistance remains a problem^9,44^. Thus, our findings suggest that in addition to BAX^41^, mutations or silencing of VDAC2 could be a potential driver of resistance to venetoclax.

## Methods

### Animal models

All mice were in an inbred C57BL/6J genetic background. All animal experiments conformed to the regulatory standards of, and were approved by, the Melbourne Health Research Directorate Animal Ethics Committee.

### Isolation of mouse liver mitochondria

Mouse livers were taken from 4 to 6 week old mice and placed in ice-cold sucrose buffer (60 mM sucrose, 210 mM mannitol, 10 mM KCl, 0.5 mM dithiothreitol, 10 mM succinate, 10 mM HEPES/KOH, pH 7.5, and 5 mM EGTA) supplemented with 1 mM phenylmethylsulfonyl fluoride and 0.1% bovine serum albumin, diced and transferred to fresh buffer in a 7 mL Wheaton tissue grinder. Aliquots were homogenized by three to five strokes. Total homogenate was diluted to 20 mL with sucrose buffer and centrifuged twice (1500 × *g*, 5 min) to remove unbroken cells. Mitochondria were then pelleted (8500 × *g*, 10 min) and washed twice with 40 mL of sucrose buffer before resuspension in 70 mM sucrose, 210 mM mannitol, 1 mM EDTA, and 10 mM HEPES/KOH, pH 7.5 at 10–30 mg/mL. Mitochondria were diluted to 1 mg/mL in MELB (100 mM KCl, 2.5 mM MgCl_2_, 100 mM sucrose, 20 mM HEPES/KOH pH 7.5, 5 mM DTT) supplemented with protease inhibitor cocktail and 4 mg/mL pepstatin A (Sigma-Aldrich) and incubated with recombinant caspase-8 cleaved BID (cBID) or a BID BH3 peptide (DIIRNIARHLAQVGDSMDRSIPPG) at 37 °C for 2 h prior to separation of soluble and membrane fractions and immunoblotting for cytochrome *c* and BAK^45,46^.

### Cell culture assays and gene targeting

MEFs isolated from embryos at embryonic day 14.5 were transformed with SV40-large T antigen^47^. MEFs were passaged in Dulbecco’s Modified Eagles Medium supplemented with 10% fetal calf serum (FCS), 55 μM 2-mercaptoethanol and 250 μM asparagine. HeLa (ATCC CCL-2), HCT116 colorectal cancer cells (a gift from R. Youle, NIH) and RS4;11 acute lymphoblastic leukemia cells (ATCC CRL-1873) were passaged in RPMI supplemented with 10% FCS. Cells were cultured at 37 °C and 10% CO_2_. The wild-type and *Vdac2*^−/−^ MEFs used in Fig. 1f were derived from 129Sv;C57BL/6 mice, otherwise all MEFs were derived from C57BL/6J inbred mice. U-251 glioblastoma cells (from A. Morokoff, Department of Surgery, University of Melbourne) were maintained in DMEM/F12 with 10% FCS.

FLAG-VDAC constructs in the vector pMX-IRES-hygromycin were retrovirally transduced into MEFs using Phoenix ecotropic packaging cells. Phoenix cells were transfected with retroviral expression constructs using FugeneX (Promega) according to manufacturer’s instructions. Virus-containing supernatants were retrieved after 48 h, passed through a 0.45 μM filter and supplemented with 4 μg/mL polybrene. Viral supernatants were then added to MEFs and cells were centrifuged at 2500 rpm for 45 min at 32 °C. Infected cells were selected on the basis of hygromycin resistance^48^.

For CRISPR/Cas9 gene editing, parental cells were lentivirally infected with constructs encoding Cas9 (*mCherry*) and doxycycline-inducible single guide RNA (*GFP*) targeting early protein coding exons of the desired gene designed using CRISPR design software (crispr.mit.edu)^49^. Following selection of transduced cells by sorting double positive cells on a FACS AriaII flow cytometer (Becton Dickinson) (Supplementary Fig. 1c), cells were treated with 1 μg/mL doxycycline hyclate (Sigma) to induce sgRNA expression. Alternatively, cells were transiently transfected with sgRNA cloned into PX458 (a kind gift from F. Zhang^50^) and individual clones were selected. Gene targeting of polyclonal populations (denoted ‘∆’) was confirmed by immunoblotting or of independent clones (denoted ‘^−/−^’) by next - generation sequencing. HCT116 *VDAC2*^−/−^ cells were generated by TALEN gene editing^14^. Sequences of all sgRNA constructs used in this study are given in Supplementary Table 4.

To test apoptotic response, cells were treated with venetoclax^7^, ABT-737^8^, A1331852^19^, ABT-263^51^, S63845 (SynMedChem)^52^, etoposide (Ebewe Interpharma), fludarabine (Sigma) or FasL (a gift from L O’Reilly). Short-term cell death response was assessed by propidium iodide uptake and flow cytometry using a FACS Calibur flow cytometer (Becton Dickinson). Long-term clonogenic potential was assessed by plating 1 × 10^5^ cells in six well plates and culturing for 5 days prior to methanol fixation and Giemsa staining of surviving cells.

All cell lines used were validated Mycoplasma negative by MycoAlert detection kit assay (Lonza).

### Genome-wide CRISPR/Cas9 library screen

MEFs constitutively expressing Cas9 were transduced with a whole-genome sgRNA library^18^ and treated with puromycin to select for a polyclonal population of sgRNA-expressing cells. Cells were treated with the BH3-mimetic ABT-737 at 250 nM (*Mcl1*^−/−^), 350 nM (*Mcl1*^−/−^*Bak*^−/−^ and *Mcl1*^−/−^*Bax*^−/−^) for 48 h. Surviving untreated and treated cells were harvested after 5 days, genomic DNA was extracted and enriched sgRNA were quantified by next-generation sequencing^49^. To identify genes whose sgRNAs had become significantly enriched in the surviving cell population, sgRNAs were ranked in descending order after calculating residuals to a lowest smoothed line fitted to log2-normalized counts for each sgRNA before and after selection. Minimum hypergeometric *P*-values were calculated from this ranked list for each gene represented in the library using an established algorithm^53^ and corrected for multiple testing.

### Analysis of subcellular fractions by PAGE and immunoblotting

Cells were permeabilized for 10 min on ice with 0.025% w/v digitonin in 20 mM Hepes (pH 7.5), 100 mM KCl, 2.5 mM MgCl_2_, and 100 mM sucrose) supplemented with complete protease inhibitors^54^. Cytosol and membrane fractions were separated by centrifugation (13,000 × *g*, 5 min, 4 °C).

For SDS-PAGE, lysates of whole cells or cellular fractions in reducing Laemmli sample buffer were electrophoresed through Tris-glycine gels (BioRad) and transferred to PVDF membrane.

For Blue native PAGE, membrane fractions were solubilized in 20 mM Bis-Tris (pH 7.4), 50 mM NaCl, 10% glycerol, 1% digitonin with or without 10 mM DTT before centrifugation at 13,000 × *g* to pellet insoluble debris. BN-PAGE loading dye (5% Coomassie Blue R-250 (Bio-Rad) in 500 mM 6-amino-hexanoic acid, 100 nM Bis-Tris (pH 7.0)) was then added to each sample. Gels were electrophoresed in anode buffer and blue cathode buffer (Invitrogen). Blue cathode buffer was replaced with clear buffer when the dye front was one-third of the way through the resolving gel^55^. Proteins were transferred to PVDF, and blots were destained in 50% methanol and 25% acetic acid prior to immunoblotting.

Membranes were blocked in 5% w/v non-fat milk in TBS-T prior to immunoblotting with antibodies raised against BAK (aa23-38, #B5897 Sigma, diluted 1:2000 in TBS-T), BAK (7D10, D.C.S. Huang, Walter and Eliza Hall Institute), BAX (49F9, D.C.S. Huang, Walter and Eliza Hall Institute), BIM (3C5, L. O’Reilly, Walter and Eliza Hall Institute) and BCL-2 (3F11, L. O’Reilly, Walter and Eliza Hall Institute), cytochrome *c* (#556433, BD Biosciences, diluted 1:2000 in TBS-T), FLAG (F3165, Sigma), GAPDH (#2118, Cell Signaling Technology), HA (#11867423001, Roche), HSP70 (W. Welch, UCSF), TOMM20 (#sc-11415, Santa Cruz Biotechnologies), TIMM44 (#HPA043052, Sigma), VDAC1 (Merck, #MABN504), VDAC2 (M.T. Ryan, Monash University, diluted 1:250 in TBS-T), VDAC3 (#55260-1-AP, Proteintech). All antibodies were diluted to 1:1000 in TBS-T unless otherwise stated. Secondary antibodies diluted 1:3000 in TBS-T were horseradish peroxidase-conjugated anti-rabbit IgG (#4010-05), anti-mouse IgG (#1010-05), and anti-rat IgG (#3010-05) (Southern Biotech). Uncropped immunoblot images are shown in Supplementary Fig. 10.

### Generation of CRISPR/Cas9 gene-targeted mice

To generate *Vdac2/Bak/Rosa* mutant mice, Cas9 mRNA (20 ng/μl) and sgRNA (10 ng/μl) were injected into the cytoplasm of fertilized one-cell stage embryos^56,57^. Twenty-four hours later, two-cell stage embryos were transferred into the uteri of pseudo-pregnant female mice. Viable offspring were genotyped by next-generation sequencing^49^.

### Blood and histological analysis of mice

Automated cell counts were performed on blood collected from the retro-orbital plexus into tubes containing EDTA (Sarstedt), using an Advia 2120 hematological analyzer (Siemens). Tissues were fixed in formalin, embedded, sectioned, and stained with hematoxylin and eosin.

### Mouse model of chemoresponse and tumor development

RS4;11 cells (1 × 10^6^) of the indicated genotypes were retrovirally transduced with a BFP-Luc luciferase reporter (pMSCV-IRES-Luciferase-BFP, gift from Dr. Zhen Xu) were engrafted subcutaneously into the flank of NOD/SCID/IL2Rγ mice by technicians blinded to the genotype of the tumor cells. After 3 weeks, mice were randomly assorted into treatment and vehicle control groups and treated for 5 days with 25 mg/kg venetoclax (ABT-199) or vehicle. To monitor tumor development, weekly 200 μL of 15 mg/mL D-luciferin potassium salt (Caliper Life Sciences) diluted in PBS was administered weekly by intraperitoneal injection. Fifteen minutes after administration of luciferin mice were anaesthetized with isoflurane inhalant and imaged using the IVIS live-imaging system (Perkin Elmer). Tumor burden was quantified by measuring the total photon flux per second emitted from the whole mouse.

To investigate tumor development, fetal liver-derived hematopoietic stem cells were harvested from mice of different genotypes (all on a C57BL/6J genetic background) at E14.5. Single cell suspensions were frozen prior to infection with retrovirus expressing *c-MYC* (pMX-IRES-GFP) in MEM supplemented with 1 mM L-glutamine, 10 mM Hepes, 1 mM sodium pyruvate, 10% (vol/vol) FCS, 50 μM β-mercatoptoethanol, and cytokines (100 ng/mL stem cell factor, 10 ng/mL IL-6, 50 ng/mL thrombopoietin, 5 ng/mL fms-related tyrosine kinase 3 ligand). Recipient C57BL/6J mice were randomly assorted into groups, lethally-irradiated (2 × 5.5 Gy, 2 h apart) and injected with cells by technicians blinded to the progenitor cell genotype. Mice were euthanized upon signs of illness (enlarged spleen or lymph nodes and weight loss). Each fetal liver was reconstituted into six lethally-irradiated mice with mice dying of irradiation toxicity (usually within 2 weeks) was censored from the analyses.

### Mass spectrometry and data analysis

Frozen livers were homogenized and solubilized in 1% Triton X-100. Proteins were resuspended in 6 M Urea, 100 mM DTT and 100 mM Tris-HCl pH7.0 and subjected to protein digestion using filter aided sample preparation^58^. Peptides in MilliQ water containing 1% acetonitrile (ACN) and 1% formic acid were analyzed by nanoflow liquid chromatography tandem-mass spectrometry (LC-MS/MS) on a nanoAcquity system (Waters, Milford, MA, USA) coupled to a Q-Exactive mass spectrometer (Thermo Fisher Scientific, Bremen, Germany) through a nanoelectrospray ion source (Thermo Fisher Scientific). Peptide mixtures were loaded on a 20 mm trap column with 180 μm inner diameter (nanoAcquity UPLC 2G-V/MTrap 5 μm Symmetry C18) at 1% buffer B, and separated by reverse-phase chromatography using a 250 mm column with 75 µm inner diameter (nanoAcquity UPLC 1.7 µm BEH130 C18) on a 120 min linear gradient from 1 to 35% buffer B (A: 99.9% Milli-Q water, 0.1% FA; B: 99.9% ACN, 0.1% FA) at a 400 nL/min constant flow rate. The Q-Exactive was operated in a data-dependent mode, switching automatically between one full-scan and subsequent MS/MS scans of the ten most abundant peaks. The instrument was controlled using Exactive series version 2.6 and Xcalibur 3.0. Full-scans (*m/z* 350–1850) were acquired with a resolution of 70,000 at 200 m/z. The 10 most intense ions were sequentially isolated with a target value of 10,000 ions and an isolation width of 3 m/z and fragmented using HCD with normalized collision energy of 27 and stepped collision energy of 15%. Maximum ion accumulation times were set to 50 ms for full MS scan and 150 ms for MS/MS. Underfill ratio was set to 2% and dynamic exclusion was enabled and set to 60 s.

For the analysis of the mitochondrial BAX/BAK complexes, mitochondria from MEFs expressing FLAG-BAK, FLAG-BAX^S184L^ or untagged BAX or BAK as controls were solubilized in 1% digitonin and immunoprecipitated with anti-FLAG-coupled sepharose. Proteins were eluted with FLAG peptide in the presence of 1% digitonin, run on BN-PAGE, and stained using Sypro Ruby (Bio-Rad) and subsequently Coomassie G-250. Bands of interest were excised, reduced with DTT (10 mM, Sigma), alkylated for 30 min with 50 mM iodoacetamide (Sigma) and digested with 600 ng of TPCK-treated trypsin Gold (Worthington) in 25 mM NH_4_HCO_3_ prior to incubation overnight at 37 °C. Peptides were then extracted in 60% ACN/0.1% FA and lyophilized to dryness using a CentriVap (Labconco) prior to reconstiution in buffer A (0.1% FA/2% ACN) ready for MS analysis. Peptides from each biological replicate were run in technical triplicate, separated by reverse-phase chromatography on a C18 fused silica column (I.D. 75 μm, O.D. 360 μm x 25 cm length) packed into an emitter tip (IonOpticks, Australia), using a nano-flow HPLC (M-class, Waters). The HPLC was coupled to an Impact II UHR-QqTOF mass spectrometer (Bruker, Bremen, Germany) using a CaptiveSpray source and nanoBooster at 0.20 Bar using acetonitrile. Peptides were loaded directly onto the column at a constant flow rate of 400 nL/min with buffer A (99.9% Milli-Q water, 0.1% formic acid) and eluted with a 90 min linear gradient from 2 to 34% buffer B (99.9% acetonitrile, 0.1% formic acid).

Mass spectra were acquired in a data-dependent manner including an automatic switch between MS and MS/MS scans using a 1.5 s duty cycle and 4 Hz MS1 spectra rate followed by MS/MS scans at 8–20 Hz dependent on precursor intensity for the remainder of the cycle. MS spectra were acquired between a mass range of 200–2000 m/z. Peptide fragmentation was performed using collision-induced dissociation (CID). The raw files were analyzed using the MaxQuant software (version 1.5.2.8)^59,60^, and extracted peaks were searched against UniProtKB/Swiss-Prot *Mus musculus* database (July 2015) containing sequences for human BAK1 and human VDAC2 and human BAX, as well as a separate reverse decoy database to empirically assess the false discovery rate using strict trypsin specificity allowing up to two missed cleavages. The minimum required peptide length was set to seven amino acids. In the main search, precursor mass tolerance was 0.006 Da and fragment mass tolerance was 40 ppm. The search included variable modifications of oxidation (methionine), amino-terminal acetylation, the addition of pyroglutamate (at N-termini of glutamate and glutamine) and a fixed modification of carbamidomethyl (cysteine). PSM and protein identifications were filtered using a target-decoy approach at a false discovery rate of 1%. Statistically-relevant protein expression changes between the samples were identified using a custom in-house designed pipeline developed in Pipeline Pilot (Biovia) and R, which utilizes the MaxQuant output files allPeptides.txt, peptides.txt and evidence.txt. A feature was defined as the combination of peptide sequence, charge and modification. Features not found in at least half the number of replicates in each group were removed. Proteins identified from hits to the reverse database and proteins with only one unique peptide were also removed. To correct for injection volume variability, feature intensities were normalized by converting to base 2 logarithms and then multiplying each value by the ratio of maximum median intensity of all replicates over median replicate intensity. Features assigned to the same protein differ in the range of intensity due to their chemico-physical properties and charge state. To further correct for these differences, each intensity value was multiplied by the ratio of the maximum of the median intensities of all features for a protein over the median intensity of the feature. Missing values were imputed using a random normal distribution of values with the mean set at mean of the real distribution of values minus 1.8 s.d., and an s.d. of 0.5 times the s.d. of the distribution of the measured intensities. The probability of differential expression between groups was calculated using the Mann–Whitney *U* test excluding any non-unique sequences and any features with modifications other than oxidation and carbamidomethylation. The output of the R function wilcox.test included the *P* value, confidence interval and ratio estimate. Probability values were corrected for multiple testing using Benjamini–Hochberg method. Cut-off lines with the function *y* = −log_10_(0.05) + *c*/(*x*−*x*_0_)^61^ were introduced to identify significantly enriched proteins. c was set to 0.2 while *x*_0_ was set to 1, representing proteins with a twofold (log2 protein ratios of 1 or more) or fourfold (log2 protein ratio of 2) change in protein expression, respectively.

### Bone marrow-derived hematopoietic precursor reconstitution

Bone-marrow was harvested from the femurs of *Vdac2*^−/−^ or age-matched wild-type control mice. Single cell suspensions were frozen at −80 °C in 90% FCS/10% DMSO until reconstitution. C57BL/6 Ly5.1 mice were lethally-irradiated with 2 × 5.5 Gy 2 h apart and reconstituted with intravenous injection of hematopoietic precursors harvested from the bone marrow of *Vdac2*^−/−^ or age-matched C57BL/6 wild-type mice in PBS. Mice were sacrificed 8 weeks post-reconstitution and blood cell analysis was performed.

### Thymocyte and splenocyte isolation and characterization

Thymus and spleen were harvested from *Vdac2*^−/−^ or age-matched wild-type control mice and single cell suspensions were generated by gentle homogenization through a 100 μm sieve. Thymocytes and splenocytes were plated at 5 × 10^4^ cells per condition in 96 well flat-bottomed plates and treated for death assay. At time of analysis, thymocytes and splenocytes were harvested and stained with AnnexinV-FITC and propidium iodide for 15 min at room temperature in AnnexinV buffer prior to analysis of viable cells (AnnexinV^-^/PI^-^) by flow cytometry. Flow cytometry data was analysed using FlowJo software.

### Platelet isolation and treatment

Platelets were isolated from peripheral blood was obtained by cardiac puncture into 0.1 volume of Aster Jandl citrate-based anticoagulant (85 mM sodium citrate, 69 mM citric acid, and 20 mg/mL glucose, pH 4.6)^62^. Platelet-rich plasma was obtained by centrifugation of the murine blood diluted in buffer A (140 mM NaCl, 5 mM KCl, 12 mM trisodium citrate, 10 mM glucose, and 12.5 mM sucrose, pH 6.0) at 125 × *g* for 8 min at room temperature. The supernatant was centrifuged at 860 × *g* for 5 min and platelets were resuspended in 10 mM Hepes, 140 mM NaCl, 3 mM KCl, 0.5 mM MgCl_2_, 10 mM glucose, and 0.5 mM NaHCO_3_, pH 7.4. 40 × 10^6^ platelets were incubated in the presence or absence of ABT-737 for 90 min at 37 °C. Death of platelets post treatment was assessed by FITC-conjugated Annexin-V binding by flow cytometry analysis.

### Statistical analysis

Unless otherwise stated, all experiments used at least three mice per experimental group. Statistical details of the experiments including statistical tests used can be found in the Figure Legends. In the cell and animal experiments statistical significance was defined as *P* < 0.05.

## Electronic supplementary material

Supplementary Information

Supplementary Data 1

Supplementary Data 2

Description of Additional Supplementary Files

## Data Availability

All the data generated and analyzed during this study are included in this published article (and its supplementary information files). The mass spectrometry proteomics data has been deposited to the ProteomeXchange Consortium via the PRIDE^63^ partner repository with the dataset identifier PXD011195.
