# Supplementary material for: VDAC2 enables BAX to mediate apoptosis and limit tumor development
Source: Nat Commun. 2018 Nov 26;9:4976. doi: 10.1038/s41467-018-07309-4 (PMC6255874; doi:10.1038/s41467-018-07309-4)
Supplement: Supplementary file 1 — Supplementary Information [file 41467_2018_7309_MOESM1_ESM.pdf]

VDAC2 enables BAX to mediate apoptosis and limit tumor development

Chin *et al*

Supplementary Information

Supplementary Fig. 1 CRISPR/Cas9 genome-wide library screen to identify novel regulators of BAX-dependent and BAK-dependent cell death.
Supplementary Fig. 2. Deletion of *Vdac2* protects from BAX-mediated apoptosis in response to BH3 mimetics.
Supplementary Fig. 3. Mitochondria lacking VDAC2 are more resistant to MOMP induced by recombinant BAX.
Supplementary Fig. 4. Characterization of the native BAX:VDAC2 and BAK:VDAC2 complexes from isolated mitochondria.
Supplementary Fig. 5. Expression of VDAC1/2 chimeras.
Supplementary Fig. 6. Efficient targeted disruption of the *Vdac2* gene using CRISPR/Cas9 in zygotes generates *Vdac2*$^{-/-}$ mice that fail to thrive.
Supplementary Fig. 7. *Vdac2*$^{-/-}$ hepatocytes have mitochondria that are resistant to MOM permeabilization and exhibit hydropic swelling.
Supplementary Fig. 8. *Vdac2* loss in primary MEFs inhibits BAX-mediated apoptosis.
Supplementary Fig. 9. *VDAC2* targeting in glioblastoma, HCT116 and RS4;11 cancer cells.
Supplementary Fig. 10. Uncropped immunoblots.

Supplementary Table 1. Enrichment of sgRNAs targeting *Bak* or *Bax* following treatment of *Mcl1*$^{-/-}$ MEFs with ABT-737.
Supplementary Table 2. Enrichment of sgRNAs targeting Bak following treatment of Mcl1$^{-/-}$ Bax$^{-/-}$ MEFs with ABT-737.
Supplementary Table 3. Enrichment of sgRNAs targeting *Bax* or *Vdac2* following treatment of *Mcl1*$^{-/-}$ *Bak*$^{-/-}$ MEFs with ABT-737.
Supplementary Table 4. Sequence of single guide RNA (sgRNA) used in this study.

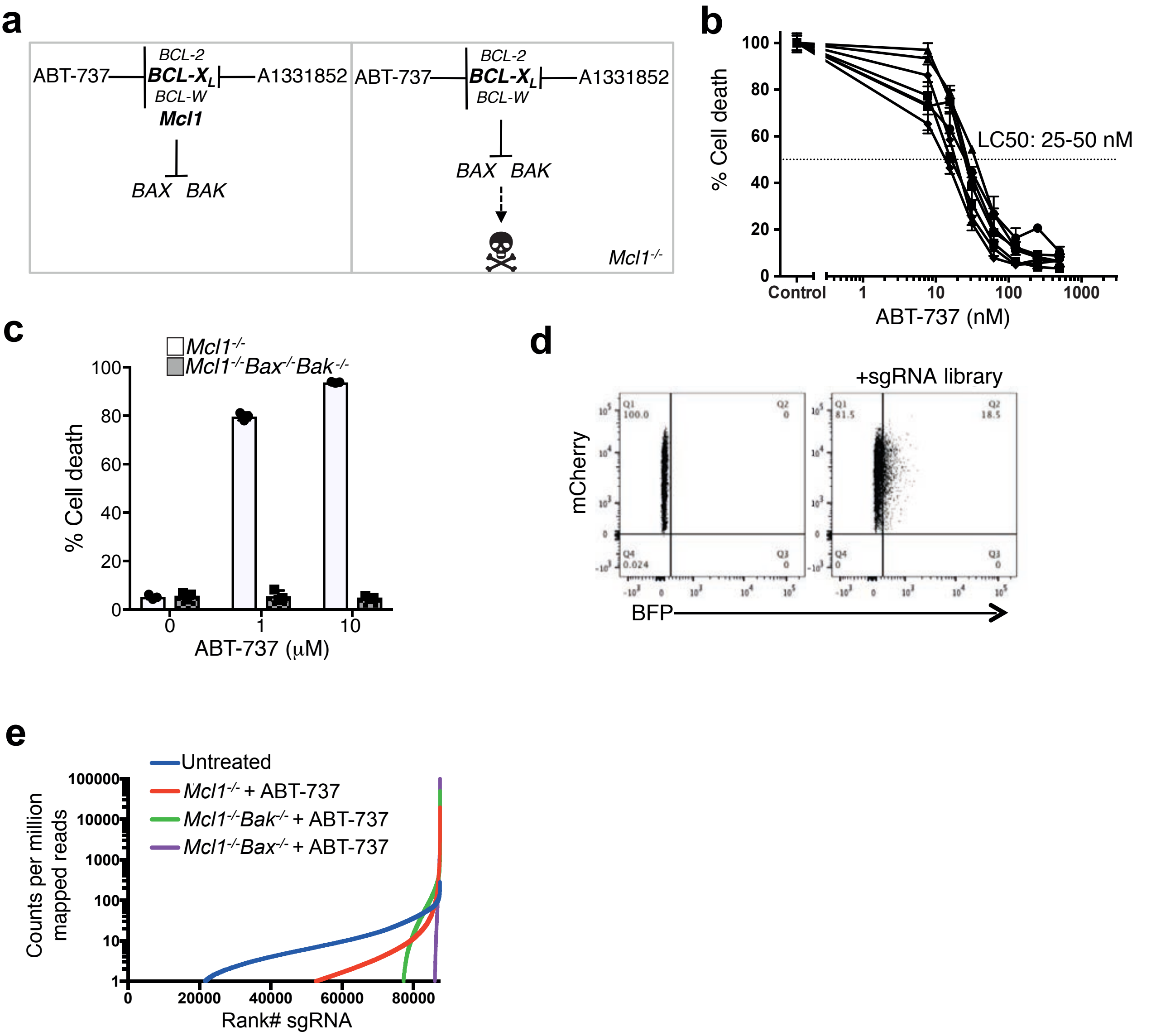

Supplementary Fig. 1 CRISPR/Cas9 genome-wide library screen to identify novel regulators of BAX-dependent and BAK-dependent cell death. (a) Schematic of sensitivity of *Mcl1*$^{-/-}$ MEFs to BH3-mimetic. The predominant pro-survival proteins in MEFs are BCL-X$_L$ and MCL1. Inhibition of BCL-2, BCL-W and BCL-X$_L$ with ABT-737 or BCL-X$_L$ with A1331852 induces cell death only in *Mcl1*$^{-/-}$ MEFs. As BCL-X$_L$ and MCL1 are the predominant pro-survivals in MEFs, inhibition of BCL-X$_L$ is sufficient to kill *Mcl1*$^{-/-}$ MEFs. **(b)** *Mcl1*$^{-/-}$ MEFs are efficiently killed by ABT-737 with a LC90 of 250 nM. Independent clones of SV40-transformed *Mcl1*$^{-/-}$ MEFs were treated with increasing does of ABT-737 and cell death assessed after 24 h by PI uptake. Data is mean +/-SEM of 3 independent experiments. **(c)** ABT-737-induced cell death is BAX/BAK-dependent. *Mcl1*$^{-/-}$ or *Mcl1*$^{-/-}$*Bax*$^{-/-}$*Bak*$^{-/-}$ MEFs were treated with the indicated dose of ABT-737 and cell death assessed by PI uptake after 24 h. Data is mean +/-SEM of 3 independent experiments. **(d)** Lentiviral transduction of expression constructs for Cas9 (mCherry) and sgRNA library (BFP). **(e)** Reduced sgRNA representation following treatment with ABT-737. In untreated control cells, almost 60,000 sgRNA were detected. Gene ontology analysis confirmed that those sgRNA that were not detected were significantly enriched for essential house-keeping genes (data not shown). Following treatment with 250 nM (for *Mcl1*$^{-/-}$) and 350 nM ABT-737 (for *Mcl1*$^{-/-}$*Bak*$^{-/-}$ and *Mcl1*$^{-/-}$*Bax*$^{-/-}$) a reduced pool of sgRNAs was recovered.

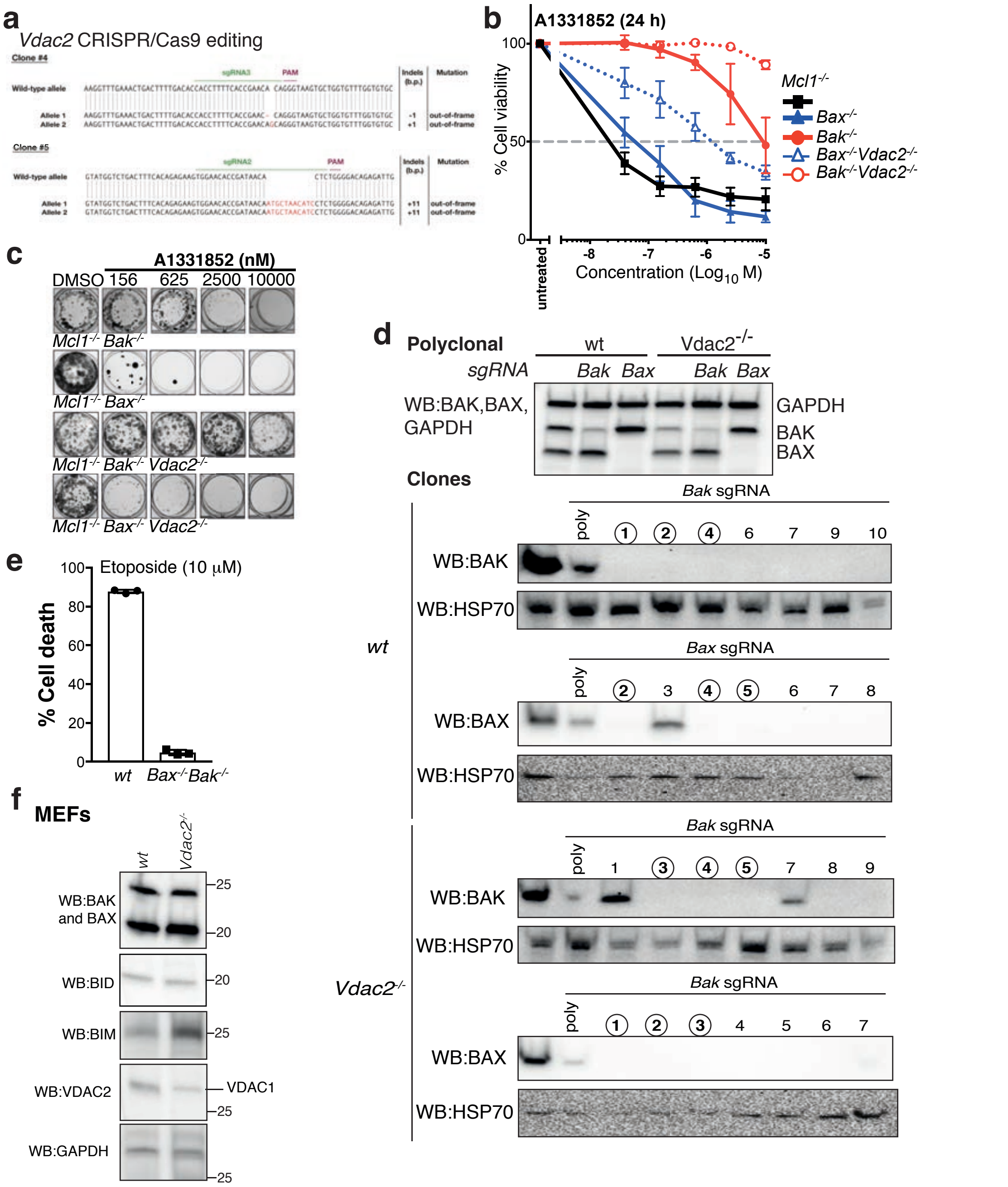

Supplementary Fig. 2. Deletion of *Vdac2* protects from BAX-mediated apoptosis in response to BH3 mimetics. (a) sgRNA targeting of *Vdac2*. *Mcl1*$^{-/-}$*Bak*$^{-/-}$ or *Mcl1*$^{-/-}$*Bax*$^{-/-}$ MEFs expressing Cas9 were transduced with sgRNA targeting *mVdac2*. Following treatment with doxycycline to induce sgRNA expression, clones were isolated and *Vdac2 was* sequenced. Examples of 2 clones with indels in *Vdac2* are shown. **(b)** Deletion of *Vdac2* protects from BAX-mediated apoptosis in response to the BCL-X$_L$ inhibition. Independent clones (*Mcl1*$^{-/-}$*Bax*$^{-/-}$*Vdac2*$^{-/-}$ and *Mcl1*$^{-/-}$*Bak*$^{-/-}$*Vdac2*$^{-/-}$) or polyclonal populations (*Mcl1*$^{-/-}$, *Bax*$^{-/-}$ and *Bak*$^{-/-}$) of MEFs were treated with A1331852 for 24 h prior to assessment of cell viability by PI exclusion. Data is mean +/- SEM of at least 3 independent experiments with 3-4 independent clones. **(c)** Deletion of *Vdac2* provides long term protection from BAX-mediated cell death in response to BCL-X$_L$ inhibition. MEFs of the indicated genotype were treated with A1331852 and colony formation assessed after 5 days. Interestingly, *Mcl1*$^{-/-}$*Bak*$^{-/-}$ MEFs, but not *Mcl1*$^{-/-}$*Bax*$^{-/-}$ MEFs, were significantly resistant to the BCL-X$_L$-specific inhibitor. This result is likely explained by the finding that BCL-2 can more efficiently inhibit BAX than BAK (Willis et al., 2005). **(d)** Validation of BAX and BAK deletion in wt and *Vdac2*$^{-/-}$ MEFs. *Bax* or *Bak* were targeted with CRISPR/Cas9 gene editing in wt or *Vdac2*$^{-/-}$ MEFs and protein expression determined by Western blotting of polyclonal populations or clones derived from single cells. The clones circled were used in the analysis of cell death in Figure 1f. **(e)** Etoposide-induced cell death is BAX/BAK-dependent. wt or *Bax*$^{-/-}$ *Bak*$^{-/-}$ MEFs were treated with etoposide (10 μM) and cell death assessed by PI uptake after 24 h. Data is mean +/- SEM of 3 independent experiments. **(f)** Absence of VDAC2 only affects BAK protein levels. Lysates of the wt or *Vdac2*$^{-/-}$ MEFs were immunoblotted as indicated.

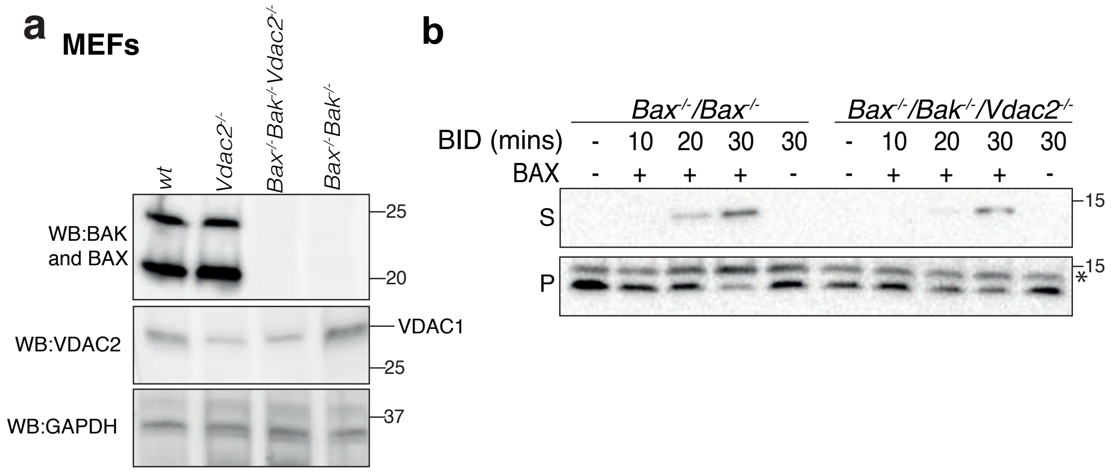

Supplementary Fig. 3. Mitochondria lacking VDAC2 are more resistant to MOMP induced by recombinant BAX. **(a and b)** Mitochondria-enriched membrane fractions from *Bax*$^{-/-}$*Bak*$^{-/-}$ or *Bax*$^{-/-}$*Bak*$^{-/-}$*Vdac2*$^{-/-}$ MEFs were incubated with recombinant BAX (50 nM) and cBID for the indicated times prior to fractionation of supernatant (S) and membrane (P) and immunoblotting for cytochrome *c*. Representative of three independent experiments. Note that the immunoblot in (a) is expanded data from Supplementary Fig. 2f.

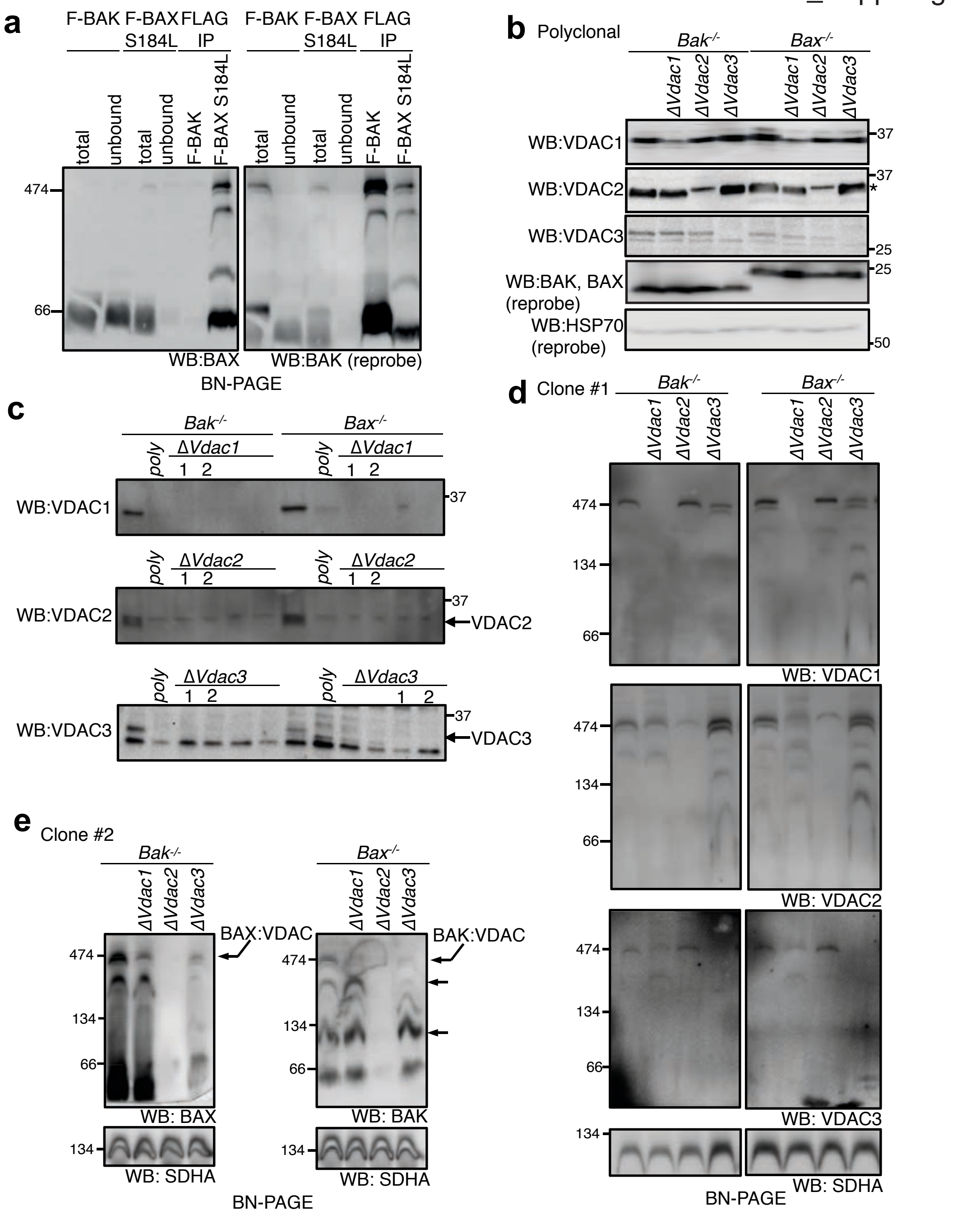

Supplementary Fig. 4. Characterization of the native BAX:VDAC2 and BAK:VDAC2 complexes from isolated mitochondria. (a) FLAG-BAX^{S184L} or FLAG-BAK stably expressed in MEFs were immunoprecipitated and purified under native conditions and assessed on BN-PAGE and immunoblotting. **(b)** Efficient deletion of VDAC isoforms. SDS-PAGE of lysates from polyclonal populations of *Bax*$^{-/-}$ or *Bak*$^{-/-}$ MEFs following CRISPR/Cas9 gene editing with sgRNA targeting *Vdac1, Vdac2* or *Vdac3* were immunoblotted with the indicated antibodies. * denotes cross-reactive VDAC1 with the anti-VDAC2 antibody. **(c)** Validation of ΔVDAC clones. Independent clones derived from the polyclonal populations shown in (b) were immunoblotted for VDAC1, VDAC2 or VDAC3. Clones #1 were used in Figure 2d, 2e and Supplementary Fig. 2d and clones #2 were used in Supplementary Fig. 2e. **(d)** Deletion of VDAC isoforms influences mitochondrial complex stability. BN-PAGE of mitochondrial fractions from clonal populations (#1) of *Bax*$^{-/-}$ or *Bak*$^{-/-}$ MEFs with CRISPR/Cas9 deletion of *Vdac1, Vdac2*, or *Vdac3* were immunoblotted with the indicated antibodies. Data is representative of two independent experiments. **(e)** Deletion of VDAC isoforms influences BAX and BAK mitochondrial complex stability. BN-PAGE of mitochondrial fractions from clonal populations (#2) of *Bax*$^{-/-}$ or *Bak*$^{-/-}$ MEFs with CRISPR/Cas9 deletion of *Vdac1, Vdac2* or *Vdac3* were immunoblotted with the indicated antibodies. Data is representative of two independent clones (see Figure 2e). Intermediate complexes indicated (arrows).

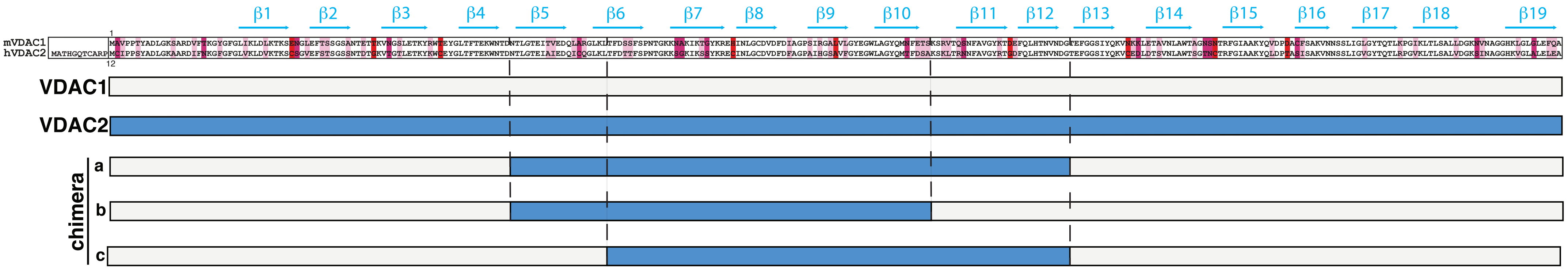

Supplementary Fig. 5. Expression of VDAC1/2 chimeras. Amino acid sequence alignment of hVDAC2 and mVDAC1 with β-sheet numbers and amino acid homology indicated.

Chin_Supp. Fig. 6

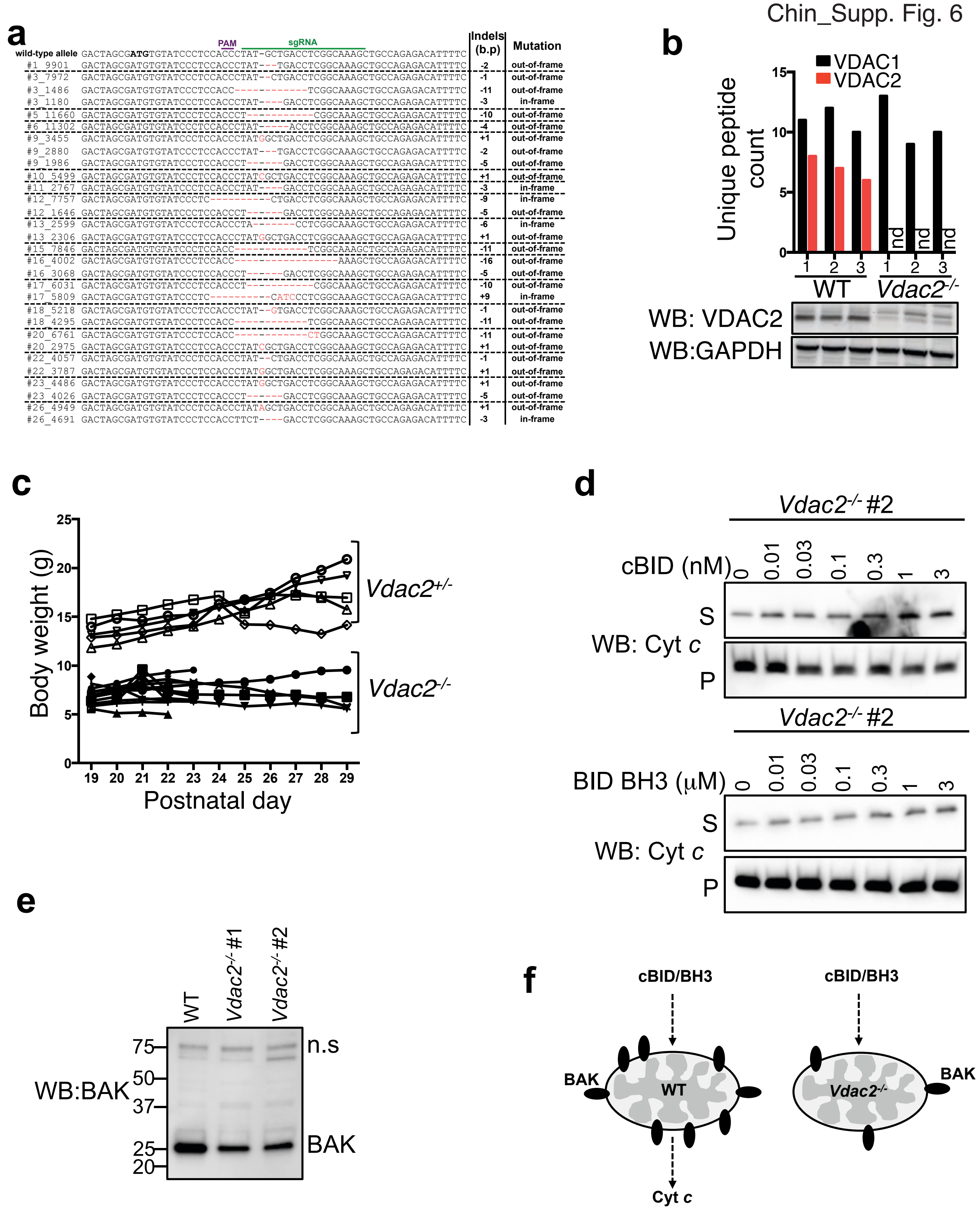

Supplementary Fig. 6. Efficient targeted disruption of the *Vdac2* gene using CRISPR/Cas9 in zygotes generates *Vdac2*$^{-/-}$ mice that fail to thrive. **(a)** Deep sequencing revealed presence of *Vdac2* indels (indicative of targeting with only the 3' exonic sgRNA) with the majority of *Vdac2*-targeted mice harboring non-sense, frameshift mutations. Regions flanking the binding site of the exonic sgRNA were sequenced. **(b)** *Vdac2*$^{-/-}$ lack of detectable VDAC2 protein. Mass spectrometry of VDAC2 or VDAC1 (bar chart) and immunoblotting for VDAC2 or GAPDH as a loading control of liver lysates from *Vdac2*$^{-/-}$ or wild-type mice. nd, not detected. **(c)** *Vdac2*$^{-/-}$, but not *Vdac2*$^{+/-}$ mice fail to gain weight after birth. *Vdac2*$^{-/-}$ (closed symbols), and *Vdac2*$^{+/-}$ (open symbols) were weighed daily. **(d)** Liver mitochondria isolated from a second *Vdac2*$^{-/-}$ mice were treated with the indicated concentration of BID BH3 peptide or cBID prior to fractionation into supernatant (S) and mitochondria-enriched membrane (P) fractions and immunoblotting for cytochrome *c*. **(e)** *Vdac2*$^{-/-}$ MLM have reduced BAK levels. Mitochondrial lysates were immunoblotted for BAK. n.s., non-specific band indicates similar loading. **(f)** Schematic representation of *Vdac2*$^{-/-}$ MLM with insufficient BAK to mediate cytochrome *c* release.

Chin_Supp. Fig. 7

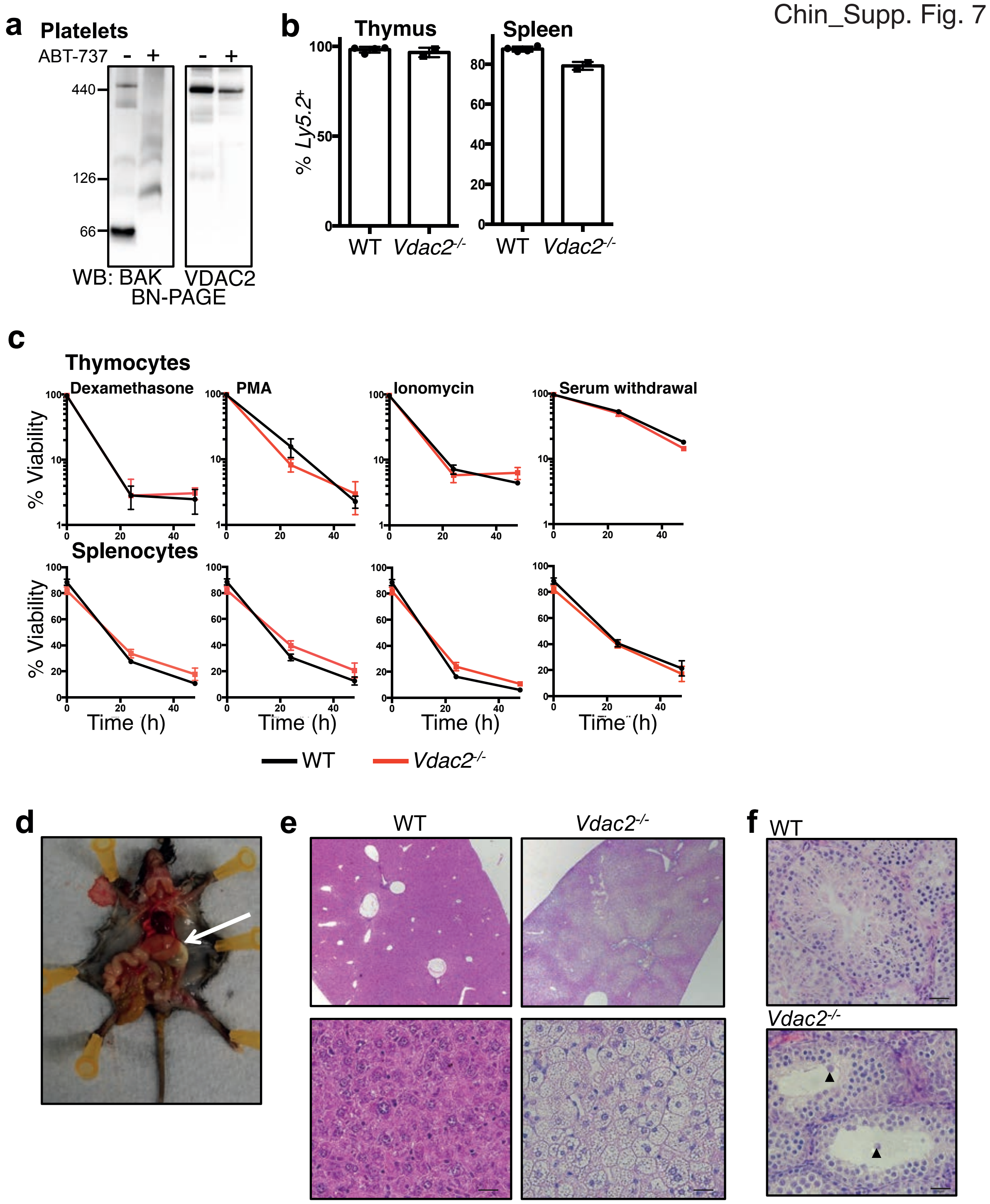

Supplementary Fig. 7. *Vdac2*$^{-/-}$ hepatocytes have mitochondria that are resistant to MOM permeabilization and exhibit hydropic swelling. (a) BAK dissociates from VDAC2 complex during platelet apoptosis. Platelets isolated from wild-type mice were incubated with ABT-737 (1.6 μM) for 90 minutes prior to BN-PAGE and immunoblotting. **(b)** Lethally-irradiated Ly5.1 *Rag*$^{-/-}$ mice were reconstituted with bone marrow-derived hematopoietic precursors from *Vdac2*$^{-/-}$ or age-matched wild-type (Ly5.2) mice. Mice were sacrificed 8 weeks post-reconstitution and the proportion of Ly5.2^{+} donor cells in the thymus and spleen were quantified. N=2-4 recipient mice. **(c)** Thymocytes and splenocytes isolated from *Vdac2*$^{-/-}$ or age-matched C57BL/6 mice were cultured in the absence of fetal calf serum (serum withdrawal) or in the presence of PMA (100 mg/ml), dexamethasone (30 nM) or ionomycin (1 mg/ml) prior to the quantitation of viable cells (AnnexinV^{-}/ PI^{-}) by flow cytometry. Data represents the mean +/-SD of three mice of each genotype **(d).** No gross defects in *Vdac2*$^{-/-}$ mice except for pale liver (*arrow*). **(e)** *Vdac2*$^{-/-}$ mice exhibit hydropic swelling of hepatocytes. Hematoxylin and eosin staining of liver sections from WT or *Vdac2*$^{-/-}$ mice show uneven distribution of staining and hepatocytes with central nuclei and clear cytoplasm. Viable non-swollen hepatocytes were largely restricted to around the portal vein. Data are representative of N=5 mice of each genotype. Scale bar represents 20 μm. **(f)** *Vdac2*$^{-/-}$ male mouse exhibits defective spermatogenesis. Histology of testicular sections from WT or *Vdac2*$^{-/-}$ mice at 5 weeks of age. Seminiferous tubules of *Vdac2*$^{-/-}$ mice are devoid of mature sperm and have giant cells (*arrowhead*) in the tubule lumen. Scale bar represents 20 μm.

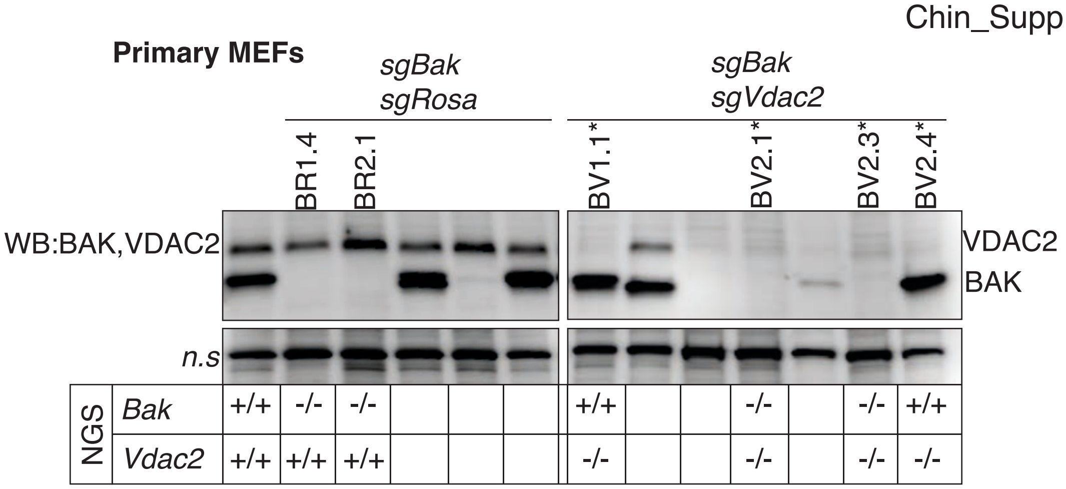

NGS													
Bak	+/+	-/-	-/-				+/+			-/-		-/-	+/+
Vdac2	+/+	+/+	+/+				-/-			-/-		-/-	-/-

Ref#	Sequence	Indels (bp)	Mutation	Genotype *Bak*
Bak	GACAAGGACCAGGTCCCCC---GAAGGTGGGCTGCGATGAGTCCCC			
BR1.4	GACAAGGACCAGGTCCCC-------GGTGGGCTGCGATGAGTCCCC<br>GACAAGGACCAGGTCCC-----GAAGGTGGGCTGCGATGAGTCCCC	-4<br>-2	out of frame<br>out of frame	-/-
BR2.1	GACAAGGACCA--------------GGTCCCCTGCGATGAGTCCCC	-11	out of frame	-/-
BV1.1*	GACAAGGACCAGGTCCCCC---GAAGGTGGGCTGCGATGAGTCCCC	-	wild-type	+/+
BV2.1*	GACAAGGACCAGGTC------------------TGCGATGAGTCCCC	-14	out of frame	-/-
BV2.3*	GACAAGGA-------------------------------TGAGTCCCC	-26	out of frame	-/-
BV2.4*	GACAAGGACCAGGTCCCCC---GAAGGTGGGCTGCGATGAGTCCCC	-	wild-type	+/+

Ref#	Sequence	Indel (bp)	Mutation	Genotype *Vdac2*
Vdac2	TGTATCCCTCCACCCTAT--GCTGACCTCGGCAAAGCTGCCAGAGA			
BR1.4	TGTATCCCTCCACCCTAT--GCTGACCTCGGCAAAGCTGCCAGAGA	-	wild-type	+/+
BR2.1	TGTATCCCTCCACCCTAT--GCTGACCTCGGCAAAGCTGCCAGAGA	-	wild-type	+/+
BV1.1*	TGTATCCCTCCA-------------CCTCGGCAAAGCTGCCAGAGA<br>TGTATCCCTCCACCCTAT-GGCTGACCTCGGCAAAGCTGCCAGAGA	-11<br>+1	out of frame<br>out of frame	-/-
BV2.1*	TGTATCCCTCCACCCTAT-GGCTGACCTCGGCAAAGCTGCCAGAGA	+1	out of frame	-/-
BV2.3*	TGTATCCCTCCACCCT-------GACCTCGGCAAAGCTGCCAGAGA<br>TGTATCCCTCCACCCTATCAGCTGACCTCGGCAAAGCTGCCAGAGA	-5<br>+2	out of frame<br>out of frame	-/-
BV2.4*	NO SEQUENCE	-	-	-/-

Supplementary Fig. 8. *Vdac2* loss in primary MEFs inhibits BAX-mediated apoptosis. Whole cell lysates from primary MEFs isolated at E14.5 targeted with *sgBak* and *sgRosa* (*BR*) or *sgBak* and *sgVdac2* (*BV*) were analyzed by immunoblotting and embryo tissue by next-generation sequencing for *Bak* and *Vdac2*. *indicates the $Bak^{-/-}$ $Vdac2^{-/-}$ embryos from which fetal livers were used for the *c-MYC*-induced AML model in Figure 6.

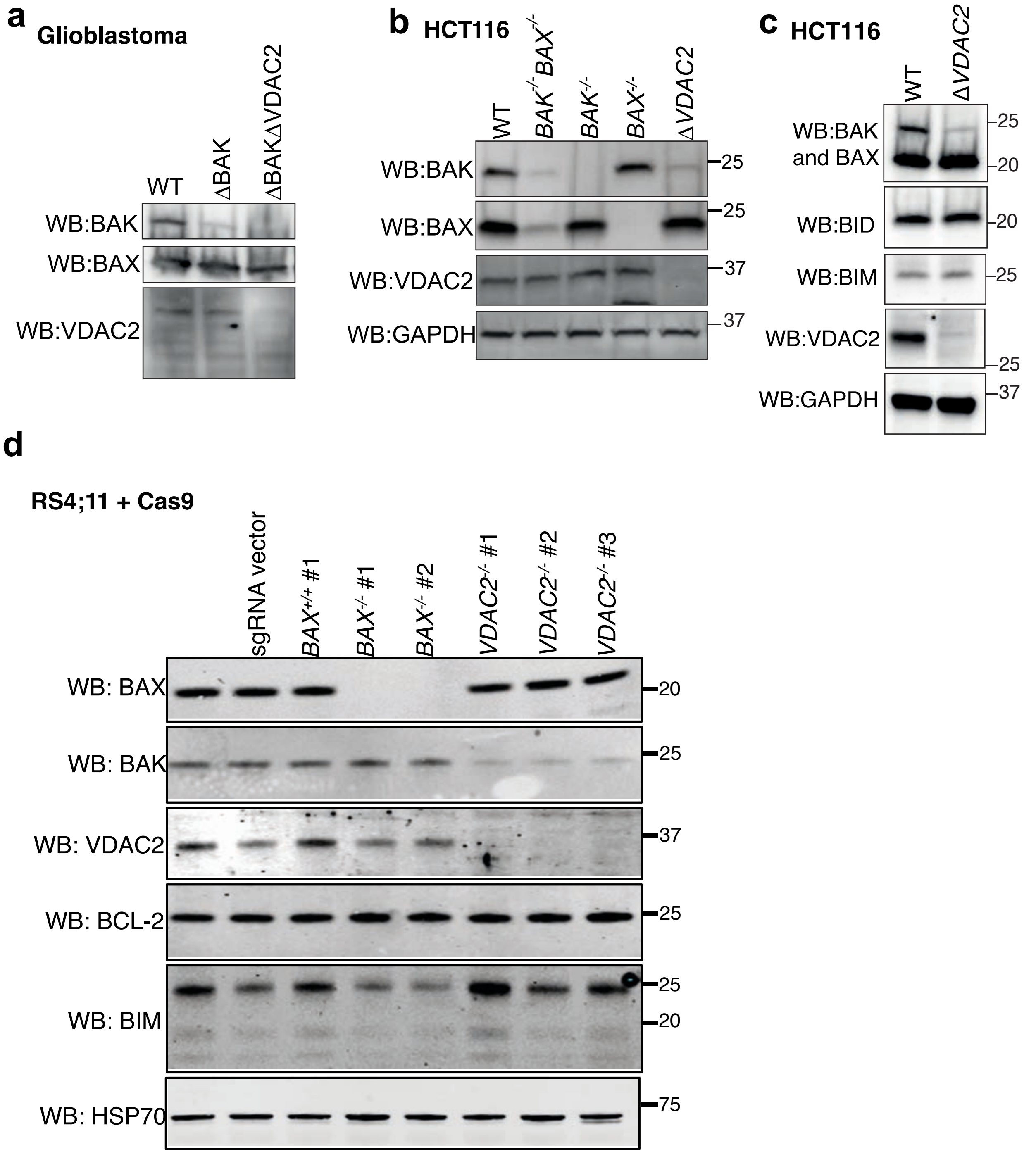

Supplementary Fig. 9. *VDAC2* targeting in glioblastoma, HCT116 and RS4;11 cancer cells. **(a)** Whole cell lysates of glioblastoma polyclonal populations engineered to lack BAK or BAK and VDAC2 by CRISPR/Cas9 gene editing were immunoblotted for the indicated proteins. **(b)** HCT116 of the indicated genotype were immunoblotted for the indicated proteins. *VDAC2*$^{-/-}$ and *BAX*$^{-/-}$ HCT116 cells were generated by TALEN-mediated and CRISPR/Cas9-mediated gene deletion in wild-type HCT116 cells respectively. N.B. *VDAC2*$^{-/-}$ HCT16 exhibited reduced BAK expression. **(c)** Absence of VDAC2 only affects BAK protein levels. Whole cell lysates of WT or Δ*VDAC2* HCT116 colorectal cells were immunoblotted as indicated. **(d)** Whole cell lysates of independent RS4;11 clones targeted for BAX or VDAC2 were analyzed for protein expression. N.B. Deletion of *VDAC2* resulted in reduced BAK protein expression.

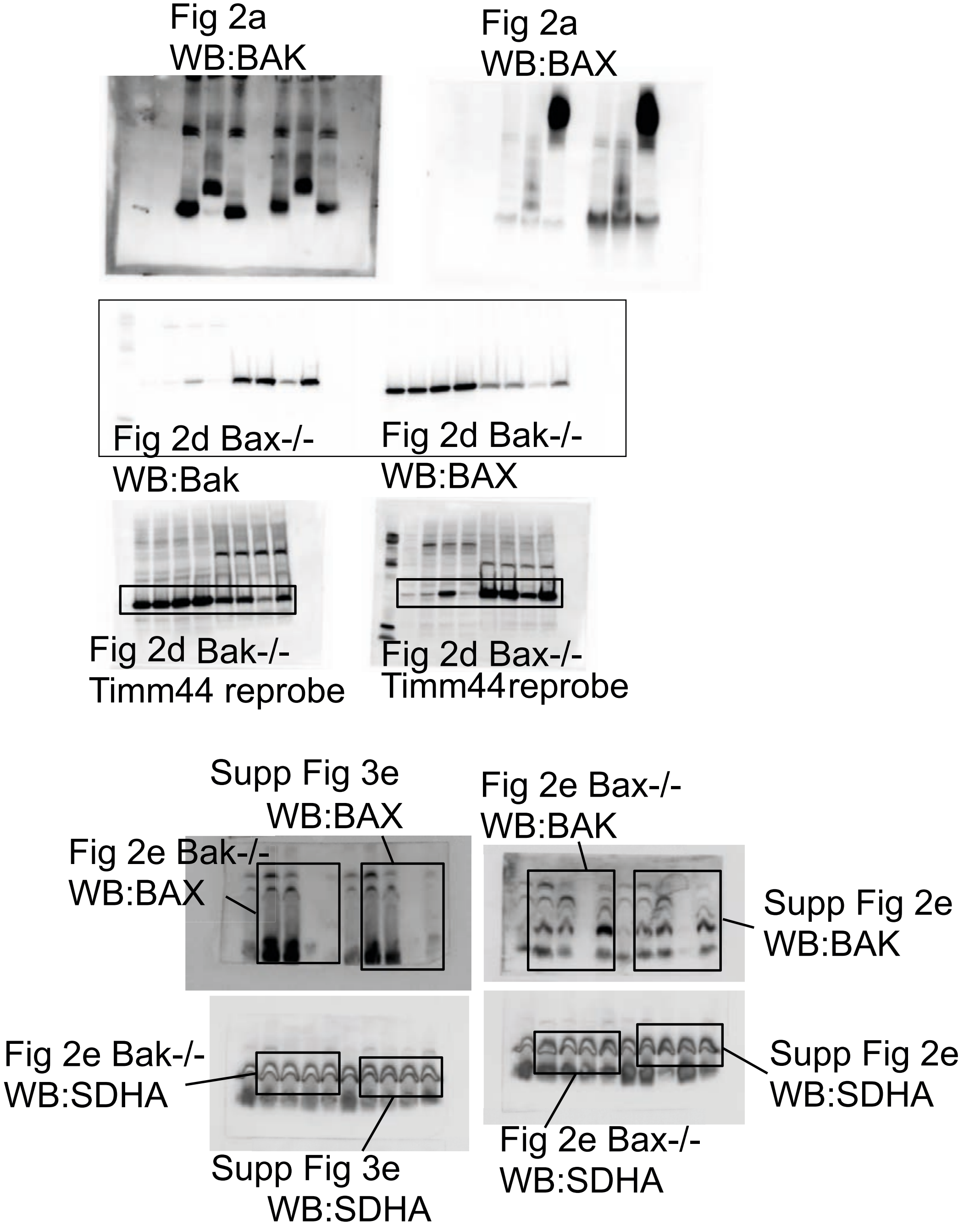

Supplementary Fig. 10. Uncropped immunoblots. Immunoblots relating to Figures 2, 3 and 4 and Supplementary Figures 2, 3, 4, 5, 6, 7, 8 and 9.

Chin_Supp Fig 10

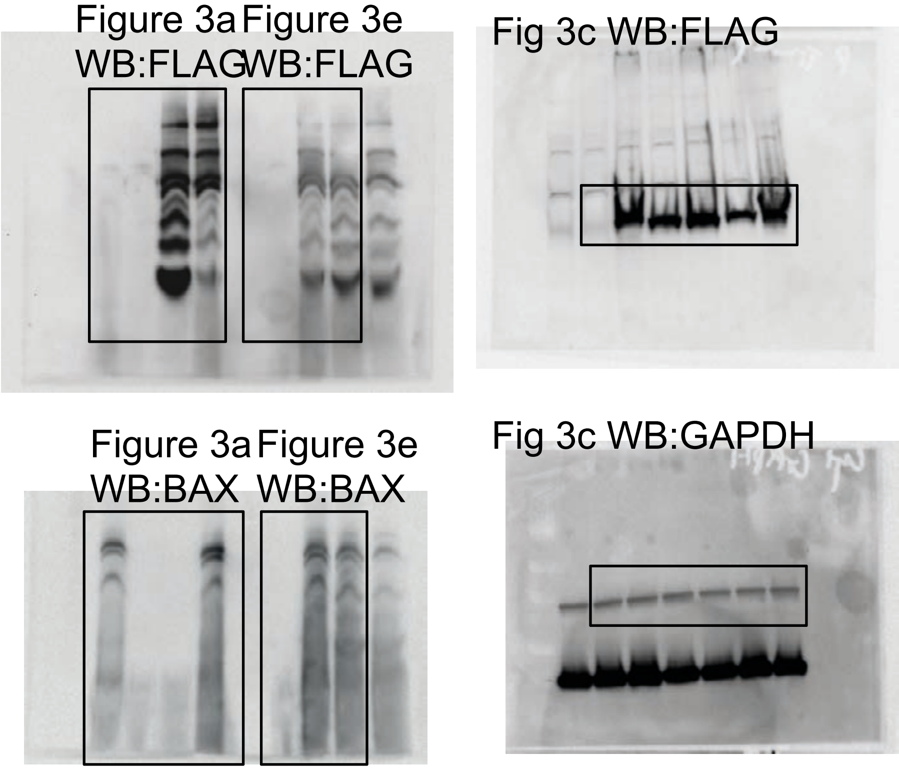

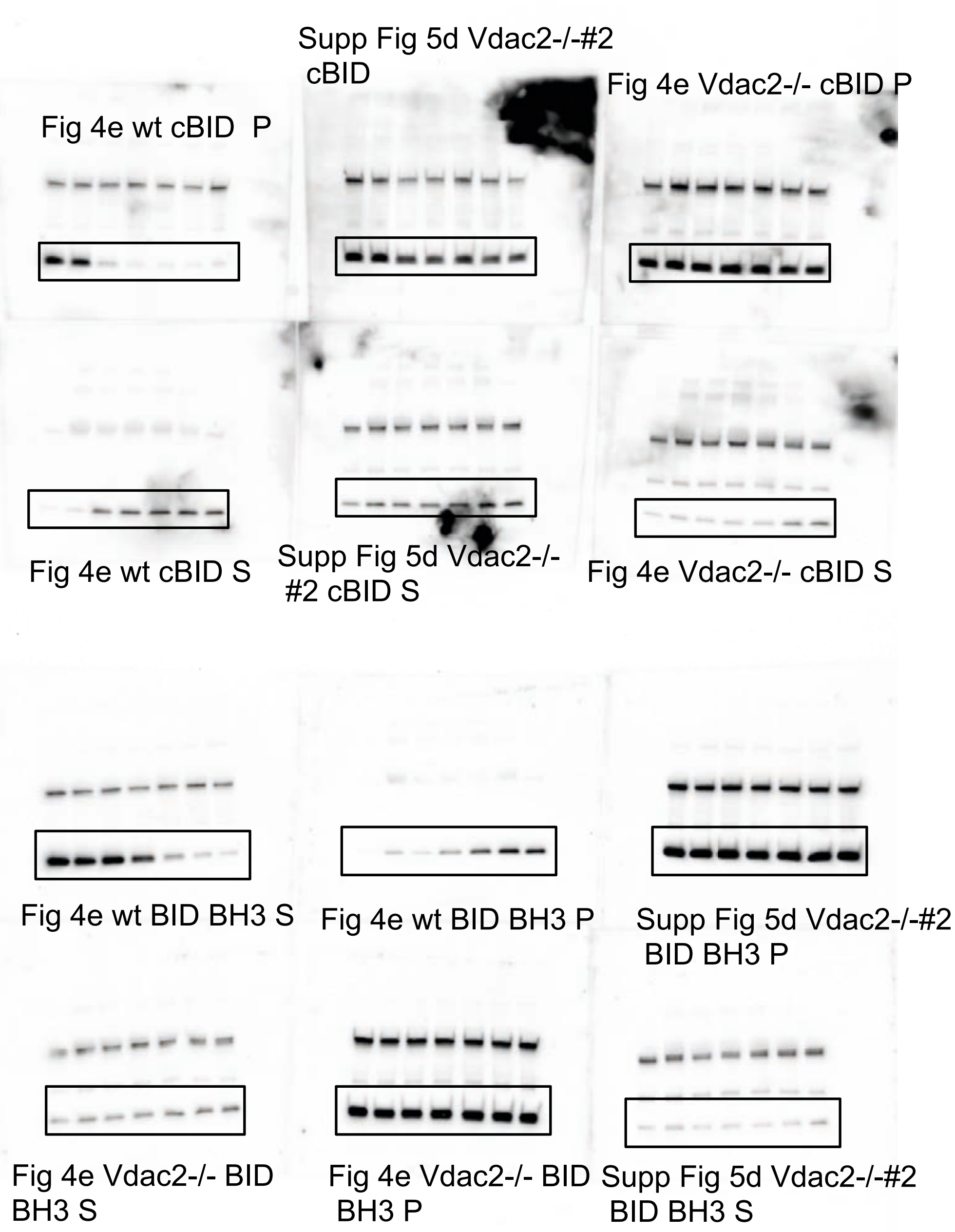
Supp Fig 5d Vdac2-/-#2 cBID
Fig 4e Vdac2-/- cBID P
Fig 4e wt cBID P
Fig 4e wt cBID S
Supp Fig 5d Vdac2-/- #2 cBID S
Fig 4e Vdac2-/- cBID S
Fig 4e wt BID BH3 S
Fig 4e wt BID BH3 P
Supp Fig 5d Vdac2-/-#2 BID BH3 P
Fig 4e Vdac2-/- BID BH3 S
Fig 4e Vdac2-/- BID BH3 P
Supp Fig 5d Vdac2-/-#2 BID BH3 S

Supp Fig 6b GAPDH

Supp Fig 6 bVDAC2

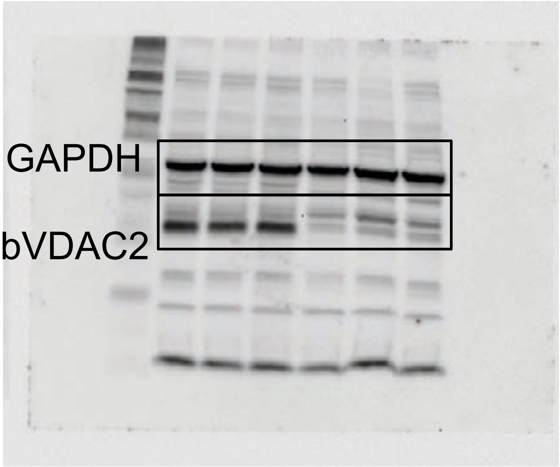

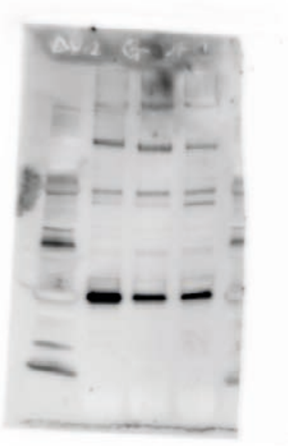

Supp Fig 7a WB: Bak

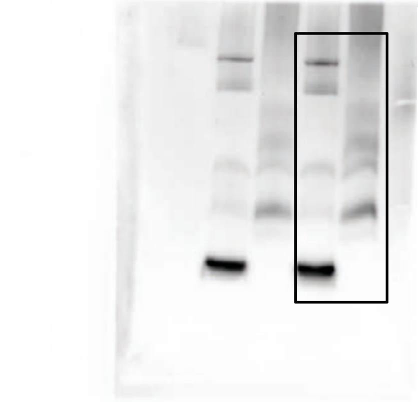

Supp Fig 7a WB: VDAC2

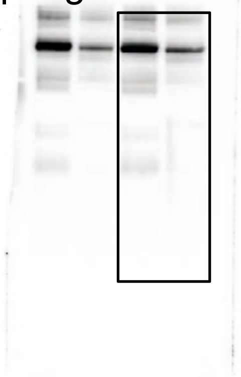

Supp Fig 8 a WB:BAK,VDAC2 (left)

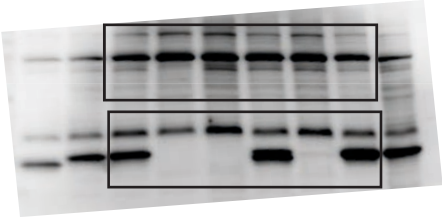

Supp Fig 8 a WB:BAK,VDAC2 (right)

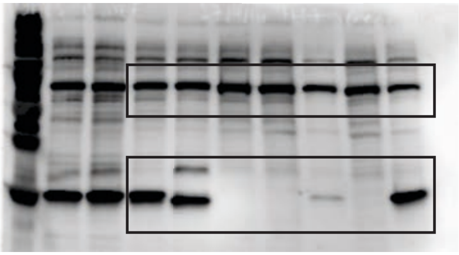

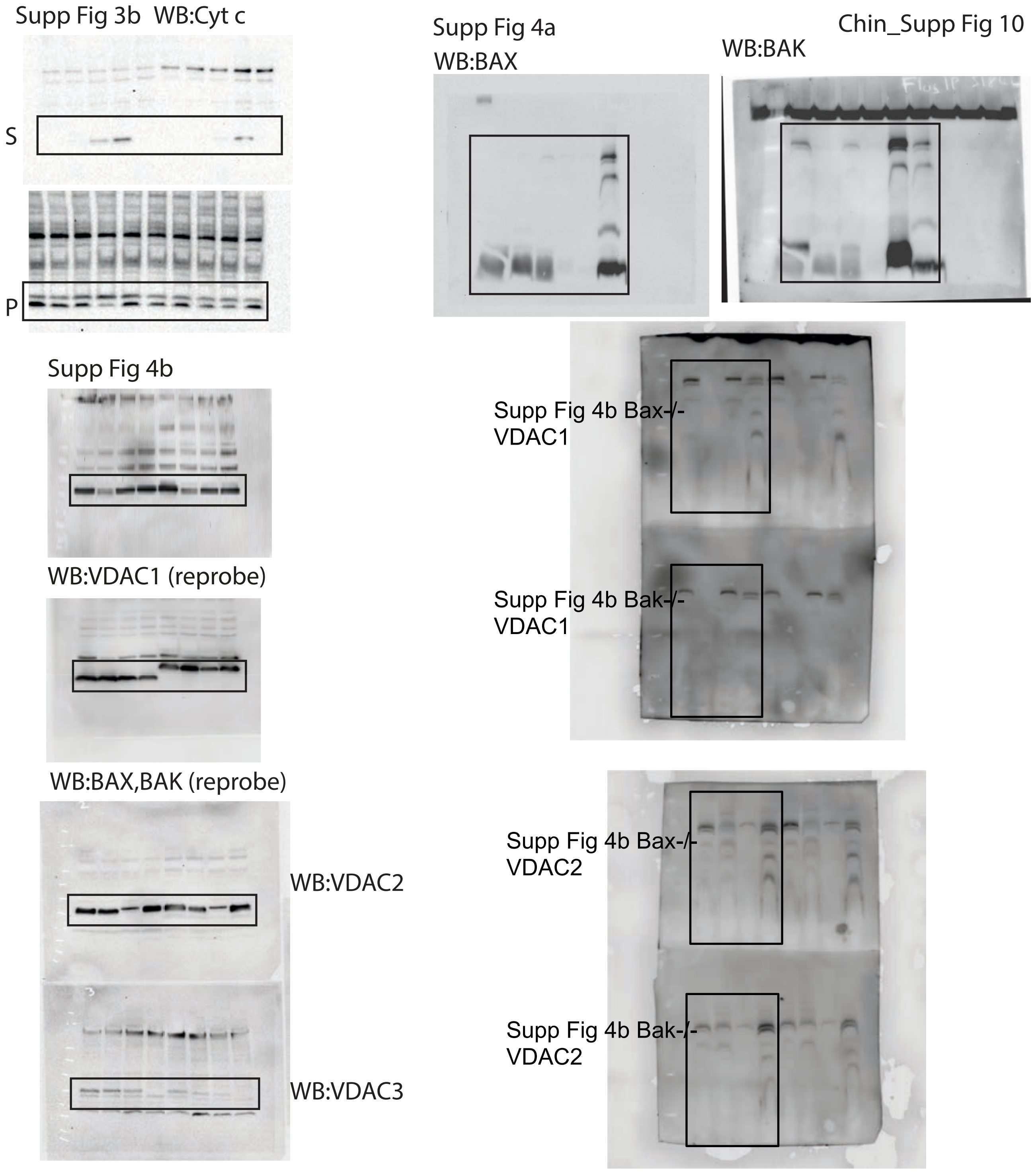
Supp Fig 3b WB:Cyt c
S
P
Supp Fig 4a
WB:BAX
Chin_Supp Fig 10
WB:BAK
Supp Fig 4b
WB:VDAC1 (reprobe)
WB:BAX,BAK (reprobe)
WB:VDAC2
WB:VDAC3
Supp Fig 4b Bax-/-
VDAC1
Supp Fig 4b Bak-/-
VDAC1
Supp Fig 4b Bax-/-
VDAC2
Supp Fig 4b Bak-/-
VDAC2

Chin_Supp Fig 10

Supp Fig 2d wt, WB:BAK

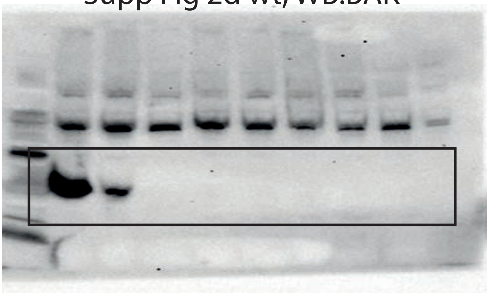

Supp Fig 2d Vdac2-/- WB:BAK

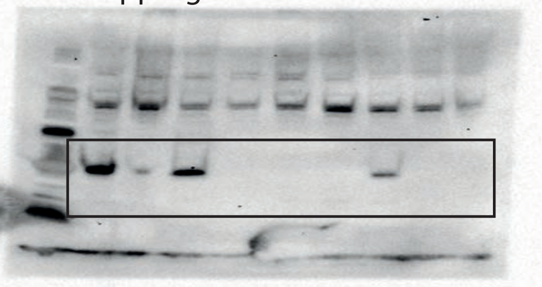

Supp Fig 2d wt, WBHSP70 (reprobe)

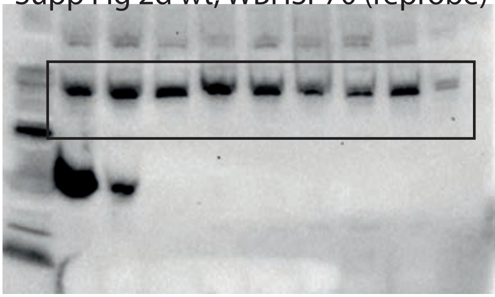

Supp Fig 2d Vdac2-/- WB:HSP70 (reprobe)

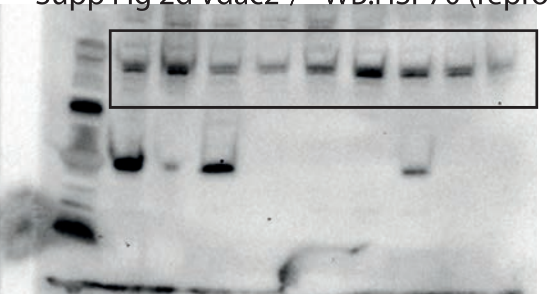

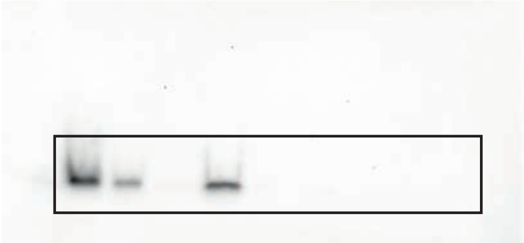

Supp Fig 2d wt, WB:BAX

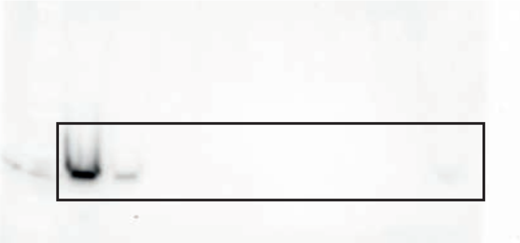

Supp Fig 2d Vdac2-/- WB:BAX

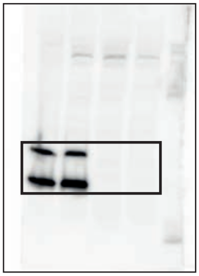

Supp Fig 2f
and Supp 3a
WB:BAX,BAK

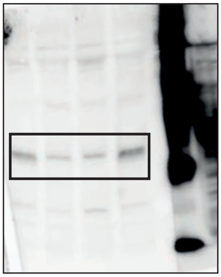

Supp Fig 2f
and Supp 3a
WB:VDAC2

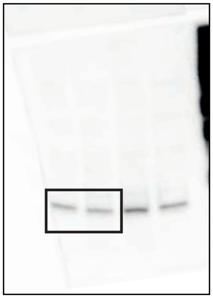

Supp Fig 2f
WB:BID

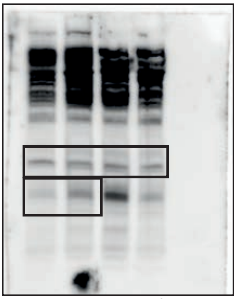

Supp Fig 2f and 3a
WB:BIM and GAPDH

Supp Fig 9a

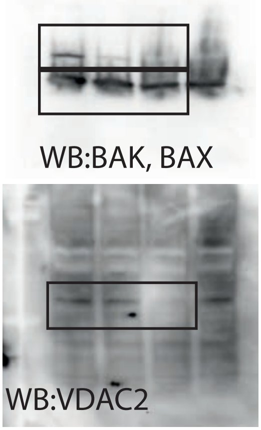

Supp Fig 9b

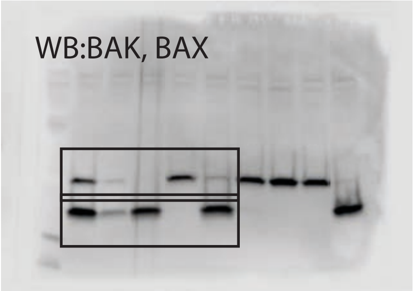

Chin_Supp Fig 10

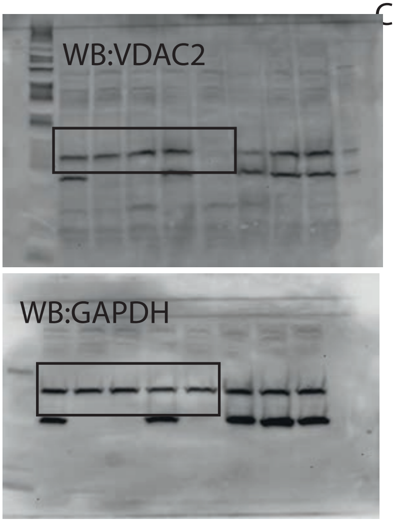

Supp Fig 9c

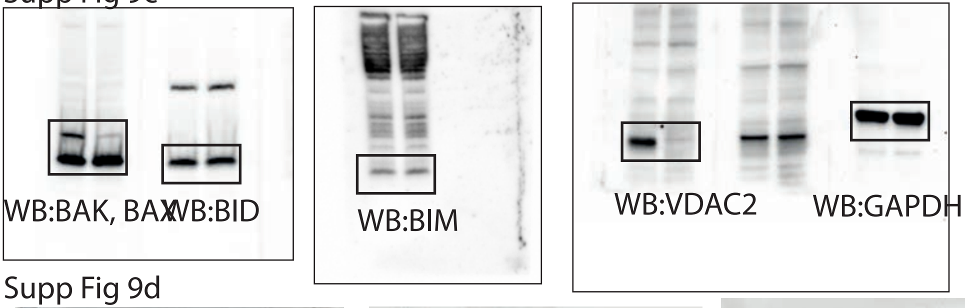

Supp Fig 9d

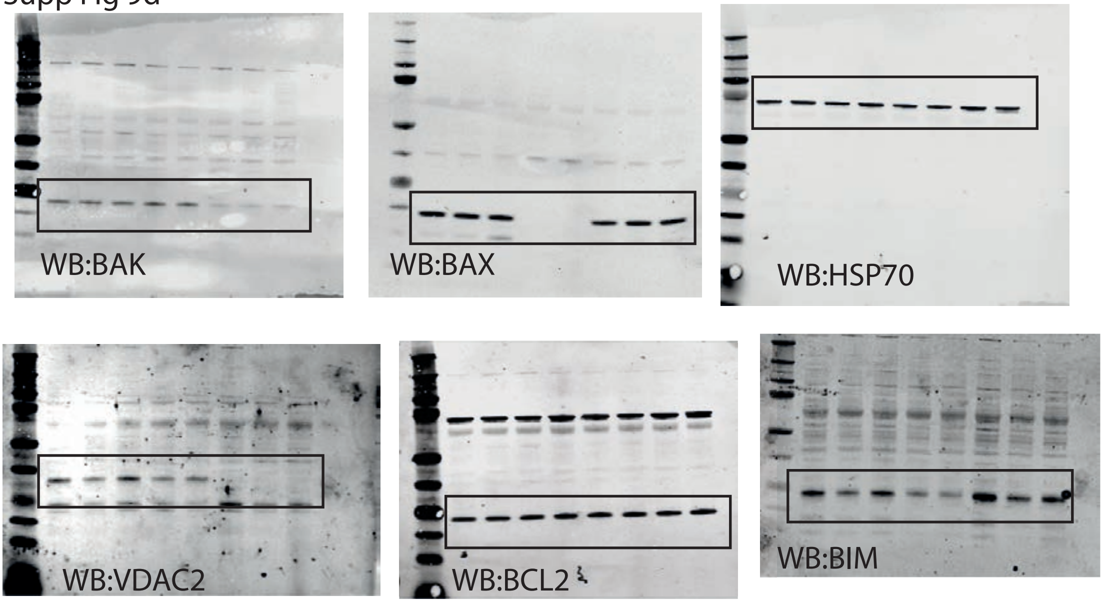

Supplementary Table 1. Enrichment of sgRNAs targeting *Bak* and *Bax* following treatment of *Mcl1*$^{-/-}$ MEFs with ABT-737 (related to Figure 1). Ranked list of the top 10 target genes based on enrichment of independent sgRNAs following treatment of *Mcl1*$^{-/-}$ MEFs with ABT-737. *Vdac2* is also shown. Data compiled from 4 independent experiments.

Mcl1$^{-/-}$

	Gene	sg1	sg2	sg3	sg4	sg5	Pval	FDR
1	***Bak1***	1	2	4	7	8	1.31E-21	2.52E-17
2	*Bax*	3	9	4703	6505	NA	5.65E-08	5.41E-04
3	*Ngly1*	4523	4672	6111	8682	8951	1.12E-05	5.50E-02
4	*6230409E13Rik*	1306	4998	6669	6785	8991	1.15E-05	5.50E-02
5	*Cyp2c50*	157	160	10718	45343	NA	1.99E-05	5.94E-02
6	*Dpp4*	36	130	10968	11591	43268	2.19E-05	5.94E-02
7	*Det1*	1016	1899	3733	4169	73055	2.48E-05	5.94E-02
8	*Kdm1b*	1210	2301	6073	9249	10516	2.51E-05	5.94E-02
9	*Olfr1298*	1356	1537	1680	32789	NA	2.79E-05	5.94E-02
10	*Dcaf11*	49	165	21413	59416	59891	3.53E-05	6.75E-02
17144	***Vdac2***	19538	39318	84609	87432	NA	0.61	0.68

Supplementary Table 2. Enrichment of sgRNAs targeting *Bak* following treatment of *Mcl1*$^{-/-}$*Bax*$^{-/-}$ MEFs with ABT-737. Ranked list of the top 10 target genes based on enrichment of independent sgRNAs following treatment of *Mcl1*$^{-/-}$*Bax*$^{-/-}$ MEFs with ABT-737. *Vdac2* is also shown. Data compiled from 2 independent experiments (related to Figure 1).

***Mcl1*$^{-/-}$ *Bax*$^{-/-}$**

	Gene	sg1	sg2	sg3	sg4	sg5	Pval	FDR
1	***Bak1***	1	2	4	7	8	1.31E-21	2.52E-17
2	*Bax*	3	9	4703	6505	NA	5.65E-08	5.41E-04
3	*Ngly1*	4523	4672	6111	8682	8951	1.12E-05	5.50E-02
4	*6230409E13Rik*	1306	4998	6669	6785	8991	1.15E-05	5.50E-02
5	*Cyp2c50*	157	160	10718	45343	NA	1.99E-05	5.94E-02
6	*Dpp4*	36	130	10968	11591	43268	2.19E-05	5.94E-02
7	*Det1*	1016	1899	3733	4169	73055	2.48E-05	5.94E-02
8	*Kdm1b*	1210	2301	6073	9249	10516	2.51E-05	5.94E-02
9	*Olfr1298*	1356	1537	1680	32789	NA	2.79E-05	5.94E-02
10	*Dcaf11*	49	165	21413	59416	59891	3.53E-05	6.75E-02
17144	***Vdac2***	19538	39318	84609	87432	NA	0.61	0.68

Supplementary Table 3. Enrichment of sgRNAs targeting *Bax* and *Vdac2* following treatment of *Mcl1$^{-/-}$Bak$^{-/-}$* MEFs with ABT-737. Ranked list of the top 10 target genes based on enrichment of independent sgRNAs following treatment of *Mcl1$^{-/-}$Bak$^{-/-}$* MEFs with ABT-737. Data compiled from 2 independent experiments (related to Figure 1).

Mcl1$^{-/-}$ Bak$^{-/-}$

	Gene	sg1	sg2	sg3	sg4	sg5	Pval	FDR
1	***Vdac2***	3	4	5	6	NA	6.16E-18	1.2E-13
2	***Bax***	1	2	7	8	NA	2.87E-17	2.8E-13
3	*Psmd2*	456	824	1224	71679	71679	2.68E-05	8.2E-02
4	*Usp47*	9	1150	1240	8425	43922	2.78E-05	8.2E-02
5	*Scpep1*	132	2142	3572	4434	17005	3.17E-05	8.2E-02
6	*Tnni2*	35	212	25412	71679	NA	3.50E-05	8.2E-02
7	*Olfr459*	197	215	71679	73011	NA	3.60E-05	8.2E-02
8	*Satl1*	901	4848	9694	10231	11301	3.60E-05	8.2E-02
9	*Fgd4*	1779	2559	3906	4659	35155	3.85E-05	8.2E-02
10	*Zfp449*	1204	2275	3724	5540	26334	7.64E-05	1.5E-01

Gene	**sgRNA sequence**
hVDAC2	ATGCTGTGCTGCTTAAGGGC
mVdac2	**G**TGGAACACCGATAACACTC*<br>*sgRNA derived from the genome-wide library and has a non-cognate G at the 5’ end to improve targeting efficiency
mVdac2	**G**CACCTTTTCACCGAACACA*<br>*sgRNA derived from the genome-wide library and has a non-cognate G at the 5’ end to improve targeting efficiency
mVdac2	GCTTTGCCGAGGTCAGCATA<br>for gene targeting in mice
mBak	ACAAGGACCAGGTCCCCCGA<br>for gene targeting in mice
mBak	TCATCGCAGCCCACCTTCGG
mBax	AGCGAGTGTCTCCGGCGAAT
mBax	AGTTTCATCCAGGATCGAGC
hBAX	CTGCAGGATGATTGCCGCCG, TCTGACGGCAACTTCAACTG
hBAK	GCATGAAGTCGACCACGAAG, GGCCATGCTGGTAGACGTGT
Control	AGCAGCAGTTCTCCGACGGT<br>for gene targeting in mice

Supplementary Table 4. Sequence of single guide RNA (sgRNA) used in this study.
