# Supplementary material for: VDAC2 enables BAX to mediate apoptosis and limit tumor development
Source: Nat Commun. 2018 Nov 26;9:4976. doi: 10.1038/s41467-018-07309-4 (PMC6255874; doi:10.1038/s41467-018-07309-4)
Supplement: Supplementary file 4 — Description of Additional Supplementary Files [file 41467_2018_7309_MOESM4_ESM.docx]

**Title: Supplementary Data 1**

Description: Complete list of protein identifications from the BAX^S184L^ complex purified under native conditions (related to Figure 2).

**Title: Supplementary Data 2**

Description: Complete list of protein identifications from the BAK complex purified under native conditions (related to Figure 2).
